# Epidemiology of adult deceased solid organ donors with positive blood cultures in the USA: a retrospective registry-based study

**DOI:** 10.1016/j.lana.2026.101638

**Published:** 2026-09-12

**Authors:** Chia-Yu Chiu, Susana Arrigain, Rocio Lopez, Edward Stenehjem, Wendy Armstrong, Jesse D. Schold

**Affiliations:** a Division of Infectious Diseases, Department of Medicine, University of Colorado, Aurora, CO, USA; b Division of Transplant Surgery, Department of Surgery, University of Colorado, Aurora, CO, USA

**Keywords:** Donor-derived infection, Blood culture contamination, *Staphylococcus aureus*, Organ procurement and transplantation network, United network for organ sharing

## Abstract

**Background:**

Organs from donors with positive blood cultures (BCx) have been shown to increase the risk of donor-derived infections. However, the percentage of BCx positivity in donors remains uncertain.

**Methods:**

We conducted a retrospective analysis using data from the United Network for Organ Sharing, examining adult donors in the U.S. from 2014 to 2024. Organisms were classified into five mutually exclusive categories: Gram-positive, Gram-negative, anaerobes, suspected contamination, and *Candida* species. The primary outcome was the annual percentage of donors with positive BCx. The secondary outcome was to identify factors associated with BCx positivity within the first two days of admission.

**Findings:**

Between 2014 and 2024, the percentage of donors with positive BCx increased, rising from 7.0% in 2014 to 13.5% in 2024. The percentage of organisms also increased: Gram-positive (3.8%–6.3%), suspected contaminants (2.9%–6.6%), and Gram-negative (0.84%–1.6%). Donors who were older, male and Black (vs. White) had increased odds of any positive BCx and coagulase-negative *Staphylococcus* (CoNS). Compared to intracranial hemorrhage/stroke, donor death from drowning had increased odds of Gram-negative (OR 3.40, 95% CI: 1.57–7.36) and anaerobic organisms (OR 5.78, 95% CI: 1.35–24.7). Donors with heavy alcohol use had increased odds of *Streptococcus pneumoniae* (OR 1.39, 95% CI: 1.08–1.79). Donors with Public Health Service risk had increased odds of *Staphylococcus aureus* (OR 2.20, 95% CI: 1.84–2.62). Compared to donation after brain death, donation after circulatory death had increased odds of *S. aureus* (OR 1.29, 95% CI: 1.10–1.52). Donors with higher social deprivation index (lower socioeconomic status) had increased odds of any positive BCx (OR 1.03, 95% CI: 1.02–1.04) and CoNS (OR 1.05, 95% CI: 1.03–1.06).

**Interpretation:**

This study presents the first national-level epidemiological data on donor blood cultures. Our findings indicate that the percentage of donors with positive BCx has increased over time, and community-acquired bacteremia is associated with donor-related factors.

**Funding:**

There was no funding source for this study.

**Research in context**

**Evidence before this study**

The Organ Procurement and Transplantation Network (OPTN)/United Network for Organ Sharing (UNOS) ad hoc Disease Transmission Advisory Committee (DTAC) is responsible for addressing donor safety and utilization, establishing classifications for donor-derived infections (DDIs), and monitoring DDIs in the United States. We searched PubMed without language restrictions using the term “(Disease Transmission Advisory Committee) and (donor-derived infection)” for publications from database inception to June 1st, 2026, and identified 23 reports. Over the past two decades, four official DTAC reports have been published, providing comprehensive case series of donor-derived infections. In addition, DTAC has released organism-specific reports focusing on selected pathogens of interest, including Cryptococcus, Coccidioidomycosis, hepatitis B (HBV), hepatitis C (HCV), and *Mycobacterium tuberculosis*. Existing theoretical and observational data suggest that donor bloodstream infection may adversely affect allograft outcomes and increase recipient mortality. Although blood cultures are routinely obtained from deceased donors, no prior study has systematically evaluated the percentage of donors with positive blood cultures at the national level. The motivation for the current study was to determine the percentage of donor blood culture positivity in the United States and to evaluate its association with donor-related factors relevant to blood culture positivity.

**Added value of this study**

Between 2014 and 2024, the percentage of deceased donors with positive blood cultures in the United States increased substantially. This increase was observed not only in organisms classified as suspected contaminants, but also among clinically relevant Gram-positive and Gram-negative bacteria. Importantly, donor blood culture positivity was significantly associated with specific donor characteristics, including cause of death, as well as sociodemographic factors.

**Implications of all the available evidence**

Donor bacteremia has increased over time, potentially due to the expansion of the donor pool (donors with public health service [PHS] risk, donors with HBV/HCV, and the opioid epidemic). Future studies should evaluate whether donor characteristics and blood culture findings can guide peri-transplant antimicrobial prophylaxis strategies. In addition, the association between specific donor bloodstream pathogens, and recipient or allograft outcomes warrants further investigation.

## Introduction

To support transplant providers in determining which infected donors may be safely utilized, the Organ Procurement and Transplantation Network (OPTN)/United Network for Organ Sharing (UNOS) ad hoc Disease Transmission Advisory Committee (DTAC) was established in the United States.^1^ The reporting of suspected or confirmed donor-derived infections (DDIs) is required under OPTN policy.^1^ Over the past decades, DTAC has published several reports describing DDIs and associated outcomes.^2^, ^3^, ^4^, ^5^ Their initial report included 3 cases of donor-derived bacteremia and 3 cases of donor-derived candidemia identified between 2005 and 2007.^2^ Subsequent reports shifted focus from bacteremia to the number of bacteria-related DDIs. The most recent report indicated that 467 donors were reported with bacterial infections, involving 1524 recipients and resulting in 230 proven or possible DDIs between 2005 and 2017.^1^

The risk of DDIs varies with the type of bacteria causing the infection. Several single-institution studies have examined graft outcomes following transplantation from donors with positive blood cultures,^1^^,^^6^, ^7^, ^8^ particularly in cases involving highly virulent organisms, such as methicillin-resistant *Staphylococcus aureus* (MRSA), vancomycin-resistant *Enterococcus*, and multidrug-resistant (MDR) Gram-negative rods. In parallel, some studies have demonstrated that the donor's cause of death may influence graft survival.^9^ These considerations raised questions about the relationship between bacteremic donors and donor factors. However, to our knowledge, this has not been evaluated by national-level data. In this study, we analyzed 10 years of OPTN data to describe the epidemiology of positive donor blood cultures and identify factors associated with culture positivity.

## Methods

### Data source

The data reported here have been supplied by UNOS as the contractor for the Organ Procurement and Transplantation Network (OPTN). The interpretation and reporting of these data are the responsibility of the author(s) and in no way should be seen as an official policy of or interpretation by the OPTN or the U.S. Government.

### Study population

We identified adult deceased donors recovered between January 1, 2014, and December 31, 2024 (with or without resulting organ transplantation). The following exclusions were applied: (1) missing date of admission or admission before January 1, 2014, (2) admission longer than 30 days, and (3) absence of blood culture data within the admission (Supplementary Figure S1).

### Sex and race and ethnicity data reporting

The organ procurement organization and the donor hospital record sex and race and ethnicity information on standard deceased donor registration forms. Data is extracted from the donor's medical record. The racial/ethnic categories with the lowest counts were combined into a category of “Other”. For detailed information on race and ethnicity data, please refer to the Supplementary Methods.

### Social deprivation

The Social Deprivation Index (SDI) is a composite, area-level metric used to quantify socioeconomic disadvantages. It ranges from 0 (least deprived) to 100 (most deprived). For detailed information please refer to the Supplementary Methods.

### Organism definition, coding validation

Organisms were classified into 5 mutually exclusive categories: Gram-positive, Gram-negative, anaerobic, suspected contamination, and *Candida* species (Supplementary Table S1). The definition of suspected contaminants (common skin commensal organisms) was adapted from the U.S. Centers for Disease Control and Prevention.^10^ Organism data was based on the descriptive text comments entered for each culture result in the database. To assess the accuracy of this coding, a random sample of culture results was selected, and one author (C.C.) manually validated the identified organisms.

### Outcome measures

The primary objective was to report the (1) percentage of donors with positive blood cultures (2) percentage of positive blood cultures among all cultures tested, and (3) percentage of positive blood cultures during admission (day 1–7). The 7-day cutoff was chosen because most donors in our data donated within 7 days of admission.

The secondary objective was to identify factors associated with blood culture positivity within 2 days of admission. The 2-day cutoff was selected based on prior literature in which early blood culture positivity has been used as a proxy for community-acquired bacteremia.^11^ However, because community acquisition could not be confirmed within the database, positivity within 2 days of admission was used as a surrogate measure. We hypothesized that these early positive cultures may be more reflective of donor-related factors than cultures identified later during hospitalization.

### Statistical analysis

We evaluated donors with culture data included in the study overall and by year, as well as donor positivity overall and within species, by year, and by day of admission. We used Poisson regression to evaluate changes in observed donor blood positivity and obtain incidence rate ratios over time. In these models all cultures for each donor were combined, and for each organism, a donor having at least one positive culture was considered positive. We used generalized estimating equations to evaluate the statistical significance of observed changes in culture positivity (using all individual cultures) over time, adjusting for repeated measures per donor and utilizing a correlation structure of compound symmetry. Supplementary Methods provided more detailed study design and data curation.

We compared donor characteristics among donors with positive blood cultures within 2 days of admission vs. those without using Wilcoxon rank-sum tests for continuous variables and Chi-square tests for categorical variables. We also compared donor characteristics among donors with at least one positive blood culture during the entire admission vs. those with none.

We performed multivariable logistic regression analyses to evaluate the factors associated with organism positivity within 2 days of admission. All cultures for each donor were combined, and for each organism, a donor having at least one positive culture was considered positive. We tested associations with the presence of any positive organism, as well as the following organisms: *S. aureus*, *Streptococcus pneumoniae*, *Streptococcus* (other species), any Gram-negative, any anaerobic, and coagulase-negative *Staphylococcus* (CoNS). The following variables were included in the logistic regression models: donor age, sex, race and ethnicity, mechanism of death, alcohol use, history of drug use, any positive of hepatitis B virus (HBV)/hepatitis C virus (HCV)/human immunodeficiency virus (HIV), Public Health Service (PHS) risk, types of deceased donors (donation after brain death [DBD] vs. donation after circulatory death [DCD]), and social deprivation index [SDI]. We used the 2019 Social Deprivation Index (SDI) developed by the Robert Graham Center (Supplementary Method).^12^ The SDI score ranges from 0 (least deprived) to 100 (most deprived) and were assigned to donors based on residential zip codes.

All analyses were performed using SAS 9.4 (SAS Institute, Cary, NC). Organism positivity was coded using R 5.4.1 (R Foundation for Statistical Computing, Vienna, Austria) with Quanteda, an R package for the quantitative analysis of textual data.^13^

### Missing data

Data were missing for the following variables: types of deceased donors (<0.05), donors have PHS risk (<0.05%), race and ethnicity (0.1%), any drug use (0.9%), heavy alcohol use (3.6%), and social deprivation index (2.1%). We used multiple imputation to create 5 imputed datasets with complete data. All model covariates were included in the imputation. Parameter estimates were pooled from models fit on each of the 5 datasets.

### Ethical statement

The study was approved by the University of Colorado Institutional Review Board (24-2064) and determined to be exempt. The requirement for informed consent was waived because the study used a de-identified dataset.

### Role of the funding source

No funding for this research.

## Results

### Cohort characteristics

Of the 123,625 recovered adult donors between 2014 and 2024, 102,485 (83%) had at least one blood culture result. These 102,485 donors had a total of 245,081 blood cultures, and the median time from admission to donation was 5 days (IQR: 4, 8) (Supplementary Table S2). On average, each donor had 2.4 blood culture results (median of 2, IQR: 1, 3).

The percentage of donors with at least one blood culture increased over time, from 5472 (72%) in 2014 to 13,881 (88%) in 2024 (Supplementary Table S2). The availability of blood culture data in the database varied significantly by region, ranging from 59% (7041/11,917) to 98% (5066/5180). Additionally, the percentage of donors having blood cultures declined over the course of hospitalization, from 36.7% on day 1 to 16.1% on day 7 (Supplementary Figure S2).

### Coding validation

The validation of the most common organisms showed that coding accuracy ranged from 92% to 99%, with sensitivity between 93% and 100%, and specificity between 89% and 99% (Supplementary Table S3).

### Positive blood culture, by year (2014–2024)

**Fig. 1:**
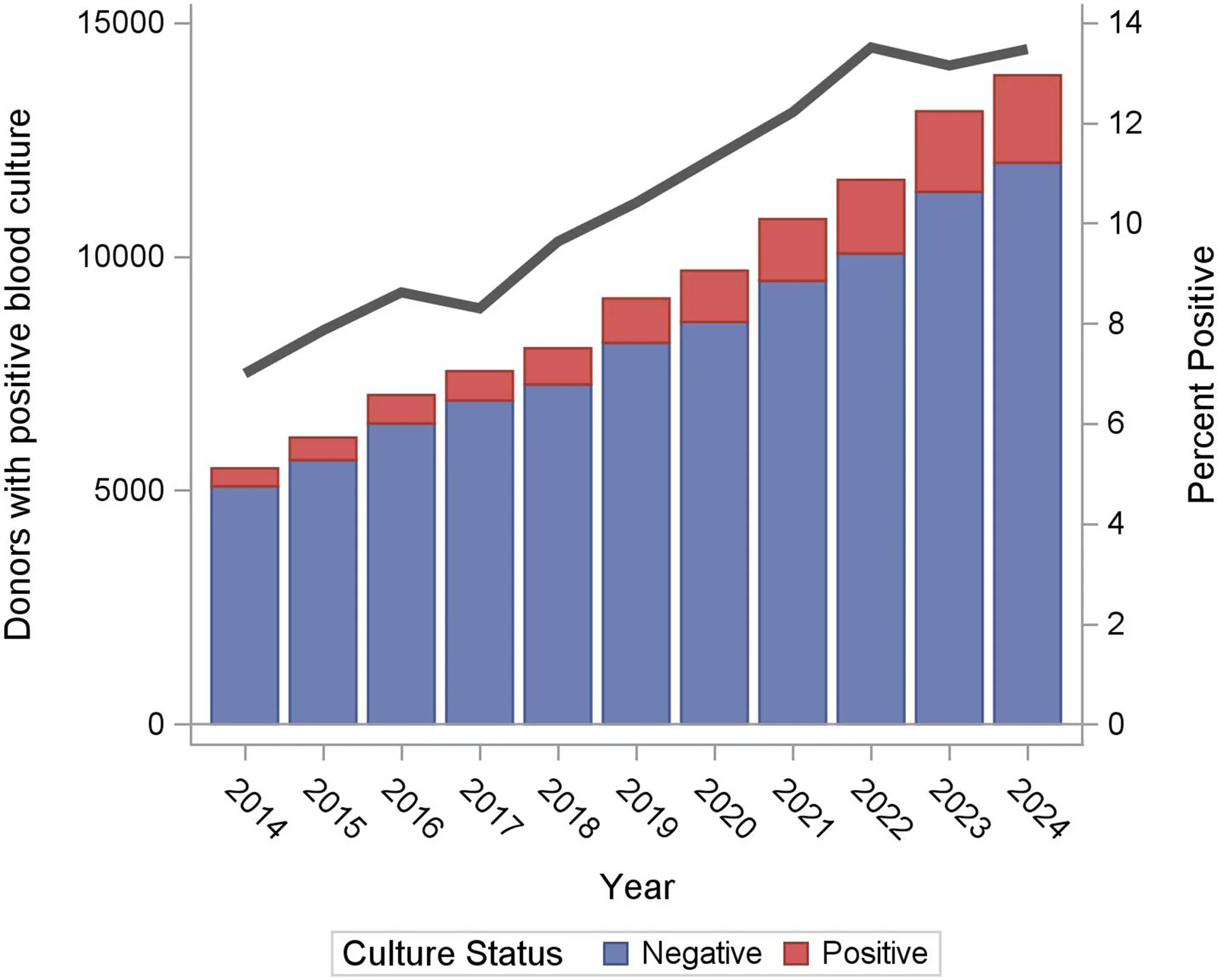
Percentage of donors with positive blood culture. The gray line represents the observed percent positive by year with corresponding y-axis on the right side of the graph. Note: unique donors (denominators).

**Fig. 2:**
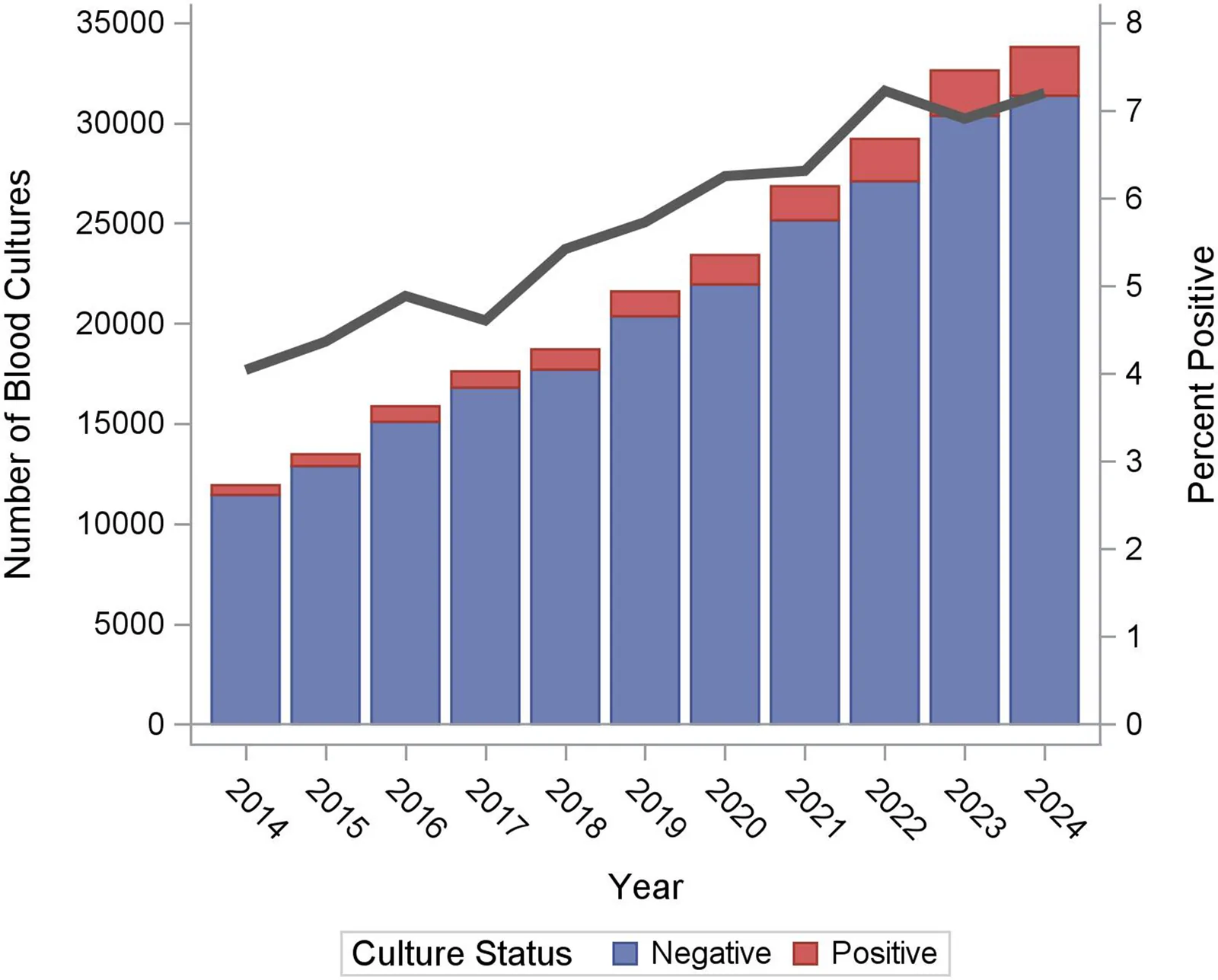
Total number of blood culture samples and percentage positive blood culture. The gray line represents the observed percent positive by year with corresponding y-axis on the right side of the graph. Note: Number of blood culture for donors (denominators). Each donor can have more than one blood culture before organ procurement.

Overall, 11,408 donors (11%) had at least one positive culture. Donors who were Black, had heavy alcohol use, and had a higher SDI had a higher percentage of blood culture positivity. Additionally, donors with cause of death being other natural causes, cardiovascular events, or drug intoxication also had a higher percentage of blood culture positivity (Supplementary Table S4). The percentage of donor blood culture positivity increased from 7% to 13.5% between 2014 and 2024, with a corresponding incidence rate ratio (IRR) of 1.93 (95% CI: 1.73–2.10) for 2024 (*p* < 0.0001) (Table 1, Fig. 1, Supplementary Table S5). The annual percentage of positive blood cultures among all cultures tested also increased, from 482 (4.0%) to 2434 (7.2%) (*p* < 0.0001) (Fig. 2). Overall, the percent of culture-level positivity was 6.1%.

**Table 1:** Percentage of donor blood culture positivity, stratified by year (2014–2024).^a^^b^

Organism	Total (N = 102,485)	2014 (N = 5472)	2015 (N = 6131)	2016 (N = 7041)	2017 (N = 7552)	2018 (N = 8043)	2019 (N = 9107)	2020 (N = 9702)	2021 (N = 10,804)	2022 (N = 11,642)	2023 (N = 13,110)	2024 (N = 13,881)
Any positive	11,408 (11.1)	383 (7.0)	482 (7.9)	607 (8.6)	627 (8.3)	775 (9.6)	948 (10.4)	1098 (11.3)	1320 (12.2)	1573 (13.5)	1724 (13.2)	1871 (13.5)
Gram-positive	5725 (5.6)	206 (3.8)	243 (4.0)	319 (4.5)	361 (4.8)	402 (5.0)	516 (5.7)	575 (5.9)	639 (5.9)	770 (6.6)	821 (6.3)	873 (6.3)
Gram-positive with no final report	2957 (2.9)	113 (2.1)	143 (2.3)	174 (2.5)	190 (2.5)	200 (2.5)	274 (3.0)	327 (3.4)	344 (3.2)	392 (3.4)	385 (2.9)	415 (3.0)
MSSA	250 (0.24)	≤10	≤10	11 (0.16)	11 (0.15)	17 (0.21)	16 (0.18)	23 (0.24)	26 (0.24)	35 (0.30)	42 (0.32)	53 (0.38)
MRSA	294 (0.29)	≤10	≤10	12 (0.17)	15 (0.20)	25 (0.31)	23 (0.25)	23 (0.24)	31 (0.29)	37 (0.32)	54 (0.41)	65 (0.47)
*Staphylococcus aureus*, unspecified	684 (0.67)	22 (0.40)	23 (0.38)	37 (0.53)	33 (0.44)	50 (0.62)	60 (0.66)	67 (0.69)	68 (0.63)	99 (0.85)	107 (0.82)	118 (0.85)
*Streptococcus agalactiae*	135 (0.13)	≤10	≤10	14 (0.20)	16 (0.21)	11 (0.14)	≤10	≤10	16 (0.15)	17 (0.15)	15 (0.11)	16 (0.12)
*Streptococcus pyogenes*	77 (0.08)	≤10	≤10	≤10	≤10	≤10	≤10	≤10	≤10	11 (0.09)	19 (0.14)	≤10
*Streptococcus pneumoniae*	417 (0.41)	21 (0.38)	13 (0.21)	21 (0.30)	27 (0.36)	26 (0.32)	45 (0.49)	31 (0.32)	29 (0.27)	61 (0.52)	68 (0.52)	75 (0.54)
*Streptococcus*, other species	1184 (1.2)	32 (0.58)	45 (0.73)	67 (0.95)	72 (0.95)	96 (1.2)	105 (1.2)	117 (1.2)	140 (1.3)	169 (1.5)	170 (1.3)	171 (1.2)
*Enterococcus faecalis*	177 (0.17)	≤10	≤10	≤10	≤10	11 (0.14)	≤10	11 (0.11)	21 (0.19)	32 (0.27)	21 (0.16)	43 (0.31)
*Enterococcus faecium*	41 (0.04)	≤10	≤10	≤10	≤10	≤10	≤10	≤10	≤10	≤10	11 (0.08)	≤10
Gram-negative	1376 (1.3)	46 (0.84)	67 (1.09)	73 (1.04)	67 (0.89)	116 (1.4)	108 (1.2)	130 (1.3)	142 (1.3)	201 (1.7)	200 (1.5)	226 (1.6)
Gram-negative with no final report	579 (0.56)	23 (0.42)	31 (0.51)	41 (0.58)	34 (0.45)	54 (0.67)	48 (0.53)	54 (0.56)	53 (0.49)	80 (0.69)	77 (0.59)	84 (0.61)
*Enterobacterales* (AmpC-producing)^c^	107 (0.10)	≤10	≤10	≤10	≤10	13 (0.16)	≤10	≤10	≤10	14 (0.12)	17 (0.13)	19 (0.14)
*Enterobacterales* (not AmpC-producing)	624 (0.61)	14 (0.26)	26 (0.42)	21 (0.30)	24 (0.32)	48 (0.60)	56 (0.61)	59 (0.61)	64 (0.59)	96 (0.82)	93 (0.71)	123 (0.89)
*Pseudomonas aeruginosa*	57 (0.06)	≤10	≤10	≤10	≤10	≤10	≤10	≤10	12 (0.11)	≤10	≤10	≤10
Anaerobic	311 (0.30)	≤10	≤10	≤10	14 (0.19)	21 (0.26)	26 (0.29)	34 (0.35)	47 (0.44)	38 (0.33)	46 (0.35)	61 (0.44)
Suspected contamination	5147 (5.0)	160 (2.9)	212 (3.5)	243 (3.5)	234 (3.1)	320 (4.0)	375 (4.1)	488 (5.0)	629 (5.8)	736 (6.3)	837 (6.4)	913 (6.6)
CoNS	3753 (3.7)	109 (2.0)	153 (2.5)	154 (2.2)	157 (2.1)	213 (2.6)	258 (2.8)	361 (3.7)	466 (4.3)	547 (4.7)	627 (4.8)	708 (5.1)
*Candida*	220 (0.21)	≤10	≤10	15 (0.21)	≤10	12 (0.15)	16 (0.18)	20 (0.21)	25 (0.23)	37 (0.32)	35 (0.27)	44 (0.32)

**Abbreviations:** CoNS, Coagulase-negative staphylococci; MSSA, Methicillin-Sensitive *Staphylococcus aureus*; MRSA Methicillin-Resistant *Staphylococcus aureus*.

a If the number of organisms is 10 or less, then it is reported as ≤10.

b The denominator is the number of donors with at least one culture. Each donor might have more than one positive blood culture, and all cultures per donor are combined.

c Amp-C producing organisms included *Citrobacter freundii, Enterobacter cloacae, Klebsiella aerogenes*.

**Fig. 3:**
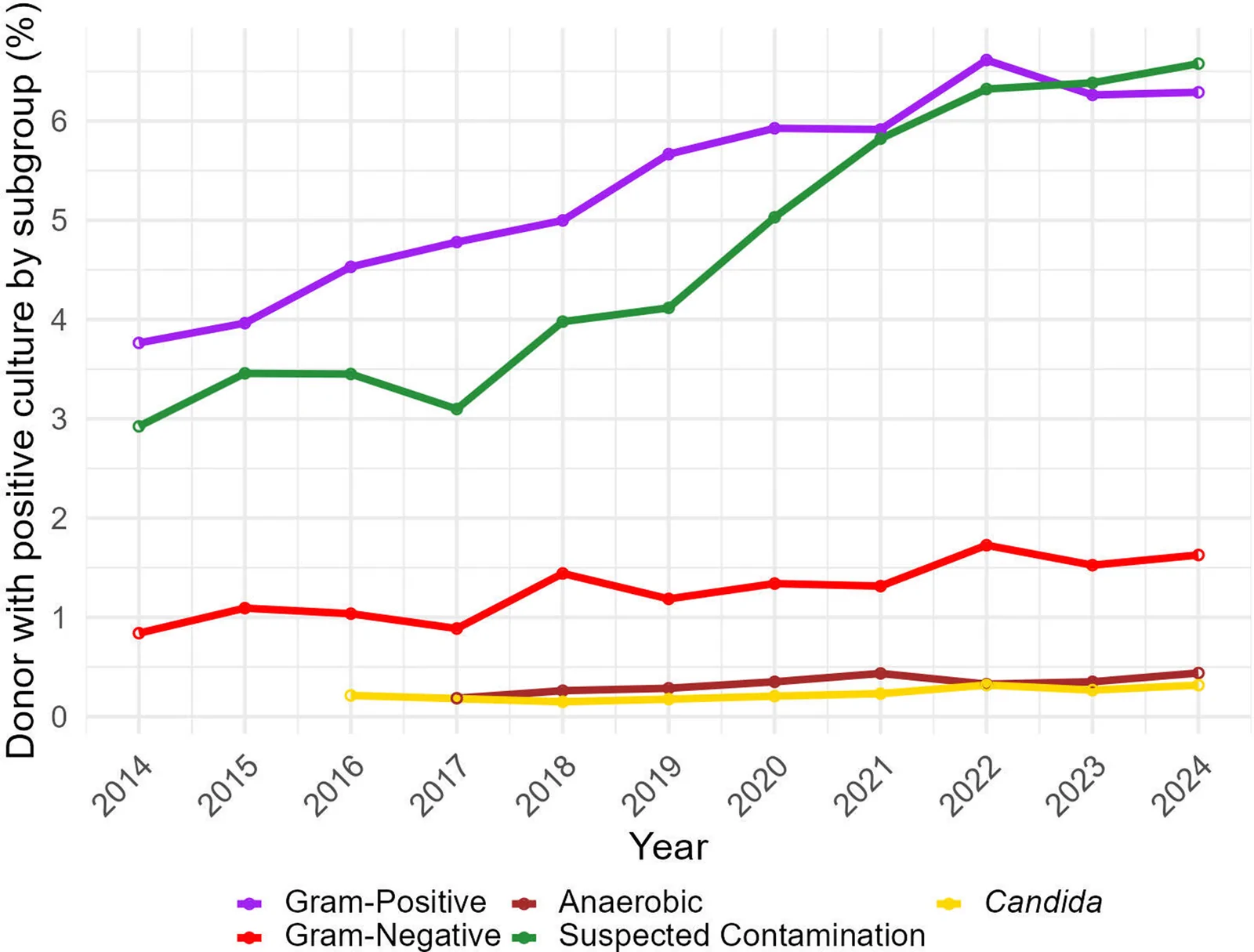
Trends of donors with positive blood culture, stratified by species. Note: Number of donors with at least one blood culture (denominators). Data points with fewer than 10 donors positive in a given year are not shown.

The average percentage of identified organisms was as follows: Gram-positive bacteria 5725 (5.6%), suspected contaminants 5147 (5.0%), Gram-negative bacteria 1376 (1.3%), anaerobes 311 (0.30%), and Candida species 220 (0.21%). The percentage of organisms also showed upward trends during this period: Gram-positive increased from 3.8% to 6.3% (IRR 1.67, 95% CI: 1.44–1.94, *p* < 0.0001), suspected contaminants from 2.9% to 6.6% (IRR 2.3, 95% CI: 1.91–2.7, *p* < 0.0001), and Gram-negative from 0.84% to 1.6% (IRR 1.94, 95% CI: 1.41–2.7, *p* < 0.0001). Anaerobes and *Candida* had too few positives in early years to analyze, but when evaluating years with consistently over 10 positives, we found increase in anaerobes from 2017 to 2024 (IRR 2.4, 95% CI: 1.33–4.2, *p* = 0.0036). In contrast, the proportion of *Candida* remained relatively stable over time (Table 1, Fig. 3, Supplementary Tables S5 and S6).

**Fig. 4:**
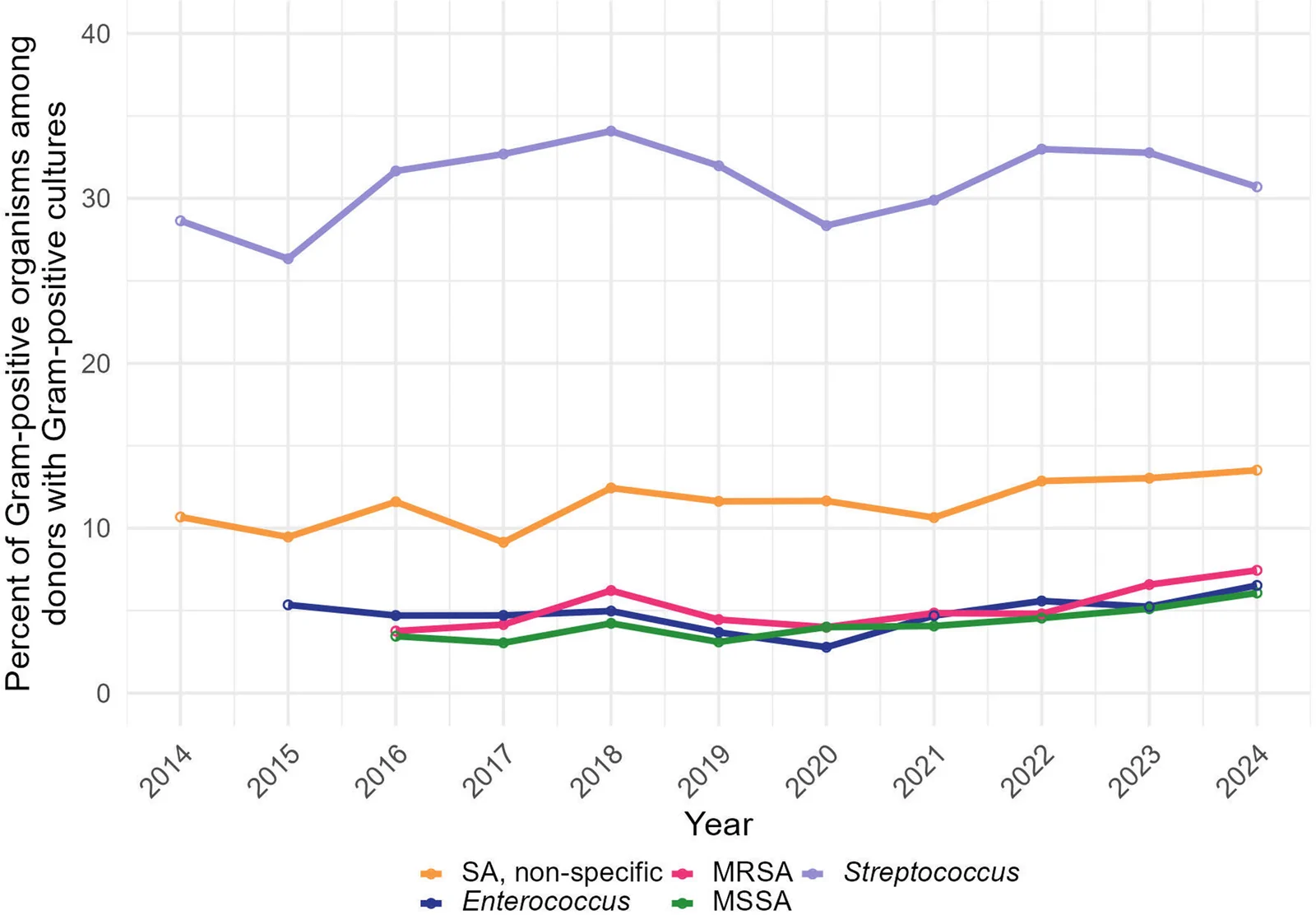
Trend of Gram-positive organisms among donors with Gram-positive blood culture. Note: Number of donors with Gram-positive organisms (denominators). Data points with fewer than 10 donors positive in a given year are not shown. Abbreviations: MSSA, methicillin-sensitive *Staphylococcus aureus*; MRSA, methicillin-resistant *Staphylococcus aureus*; SA, *Staphylococcus aureus*.

**Fig. 5:**
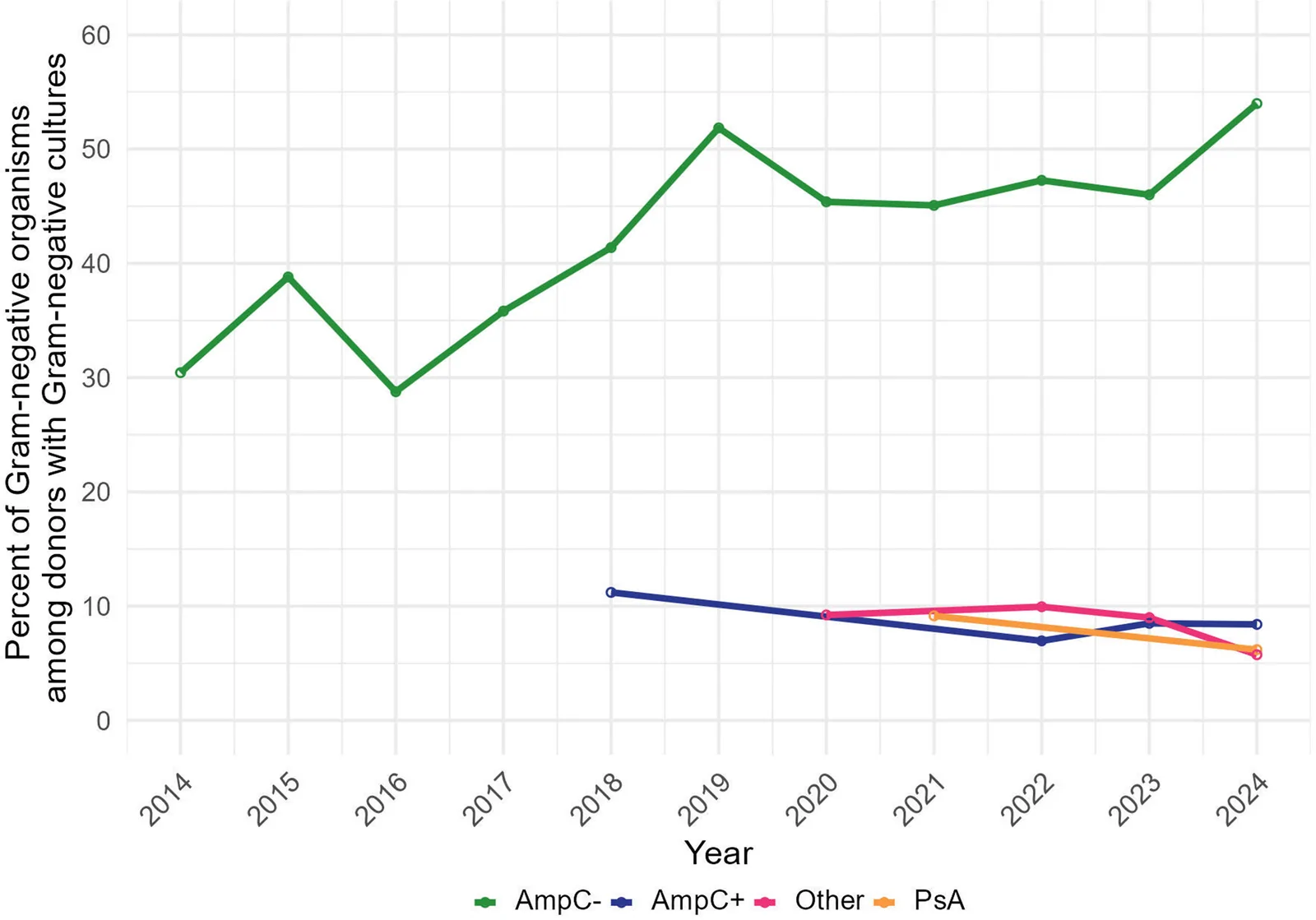
Trend of Gram-negative organisms among donors with Gram-negative blood culture. Note: Number of donors with Gram-negative organisms (denominators). Data points with fewer than 10 donors positive in a given year are not shown. Abbreviations: AmpC, AmpC beta-lactamase; PsA, *Pseudomonas aeruginosa*.

**Fig. 6:**
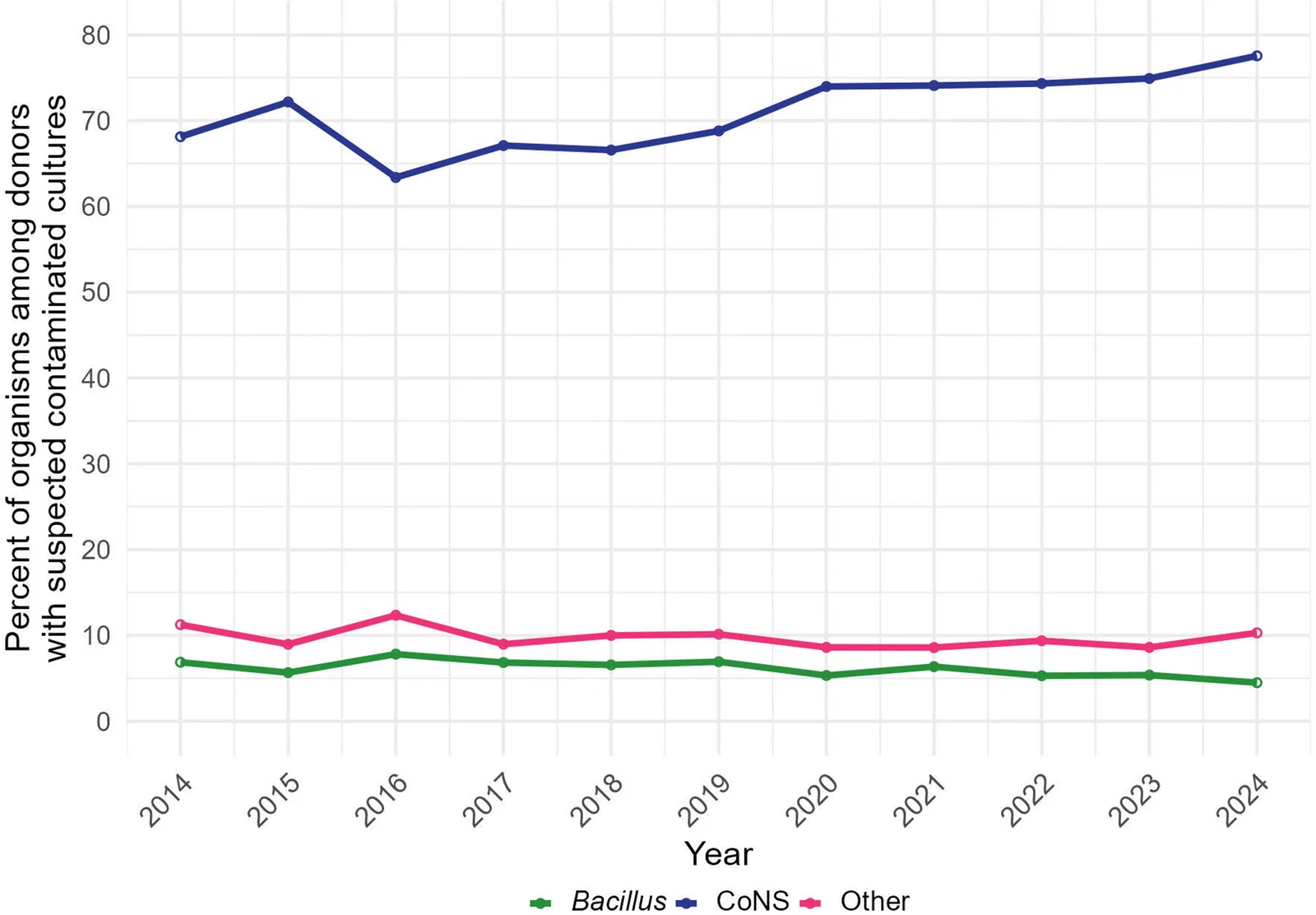
Trend of suspected contamination organisms among donors with suspected contaminated blood culture. Note: Number of donors with suspected contamination organisms (denominators). Data points with fewer than 10 donors positive in a given year are not shown. Abbreviation: CoNS, Coagulase-negative staphylococci.

Among 5725 donors with Gram-positive organisms, 2957 (52%) lacked a final culture report. Among those with final data available, *Streptococcus* species were the most common (including 1184 other *Streptococcus*, 417 *S. pneumoniae*, 135 *Streptococcus agalactiae*, 77 *Streptococcus pyogenes*), followed by *S. aureus* (including 684 unspecified *S. aureus*, 250 methicillin-susceptible *S. aureus* [MSSA], 294 methicillin-resistant *S. aureus* [MRSA]). The type of Gram-positive organisms identified in donors remained relatively stable (Fig. 4). Among 1376 donors with Gram-negative organisms, 579 (42%) lacked a final culture report. The percentage of non-AmpC-producing *Enterobacterales* increased from 14 (30%) in 2014 to 122 (54%) in 2024 (Fig. 5). Of 5147 donors with suspected contamination organisms, 3753 (73%) had coagulase-negative staphylococci (CoNS). The percentage of donors with CoNS was 68% (N = 109) in 2014 and 77% (N = 708) in 2024 (Fig. 6).

### Positive blood culture, by admission day (day 1–day 7)

The percentage of blood culture-level positivity during admission decreased from 11.6% on day 1 to 5.0% on day 7 (Supplementary Figure S3). On day 1, all categories, except *Candida*, had their highest donor-level positivity: Gram-positive bacteria (8.0%), suspected contaminants (6.8%), Gram-negative bacteria (1.5%), anaerobes (0.57%), and *Candida* species (0.14%). The frequency of *S. aureus* remained stable from day 2 to day 7. The frequency of CoNS was highest on day 1 (4.9%) and remained stable from day 2 through day 7 (<2.0%). In contrast, Gram-negative organisms showed an upward trend from day 5 to day 7, increasing from 0.74% to 1.2%. *Candida* species showed an increasing trend from day 2 through day 7, rising from 0.05% to 0.20% (Table 2, Supplementary Table S7).

**Table 2:** Donor blood culture results, stratified by day of admission (days 1–7).^a^^b^

Organism	Day of admission						
	1 (N = 37,578)	2 (N = 31,795)	3 (N = 22,968)	4 (N = 17,603)	5 (N = 12,448)	6 (N = 8706)	7 (N = 6367)
Any positive	5716 (15.2)	1586 (5.0)	1213 (5.3)	1049 (6.0)	728 (5.8)	508 (5.8)	406 (6.4)
Gram-positive	2991 (8.0)	872 (2.7)	635 (2.8)	516 (2.9)	363 (2.9)	244 (2.8)	172 (2.7)
Gram-positive with no final report	1287 (3.4)	453 (1.4)	334 (1.5)	278 (1.6)	180 (1.4)	124 (1.4)	84 (1.3)
MSSA	88 (0.23)	36 (0.11)	49 (0.21)	41 (0.23)	31 (0.25)	17 (0.20)	11 (0.17)
MRSA	146 (0.39)	52 (0.16)	55 (0.24)	41 (0.23)	27 (0.22)	21 (0.24)	20 (0.31)
*Staphylococcus aureus*, unspecified	295 (0.79)	120 (0.38)	101 (0.44)	97 (0.55)	88 (0.71)	42 (0.48)	36 (0.57)
*Streptococcus agalactiae*	81 (0.22)	22 (0.07)	≤10	≤10	≤10	≤10	≤10
*Streptococcus pyogenes*	53 (0.14)	≤10	≤10	≤10	≤10	≤10	≤10
*Streptococcus pneumoniae*	332 (0.88)	42 (0.13)	20 (0.09)	≤10	≤10	≤10	≤10
*Streptococcus*, other species	807 (2.1)	160 (0.50)	71 (0.31)	52 (0.30)	33 (0.27)	27 (0.31)	13 (0.20)
*Enterococcus faecalis*	88 (0.23)	18 (0.06)	17 (0.07)	17 (0.10)	≤10	≤10	≤10
*Enterococcus faecium*	17 (0.05)	≤10	≤10	≤10	≤10	≤10	≤10
Gram-negative	580 (1.5)	143 (0.45)	129 (0.56)	137 (0.78)	92 (0.74)	73 (0.84)	74 (1.2)
Gram-negative with no final report	243 (0.65)	66 (0.21)	57 (0.25)	56 (0.32)	38 (0.31)	25 (0.29)	30 (0.47)
*Enterobacterales* (AmpC-producing)^c^	23 (0.06)	≤10	13 (0.06)	15 (0.09)	≤10	≤10	15 (0.24)
*Enterobacterales* (not AmpC-producing)	283 (0.75)	61 (0.19)	59 (0.26)	57 (0.32)	35 (0.28)	33 (0.38)	24 (0.38)
*Pseudomonas aeruginosa*	17 (0.05)	≤10	≤10	≤10	≤10	≤10	≤10
Anaerobic	214 (0.57)	41 (0.13)	12 (0.05)	11 (0.06)	≤10	≤10	≤10
Suspected contamination	2567 (6.8)	612 (1.9)	485 (2.1)	434 (2.5)	290 (2.3)	199 (2.3)	164 (2.6)
CoNS	1824 (4.9)	408 (1.3)	344 (1.5)	330 (1.9)	210 (1.7)	141 (1.6)	126 (2.0)
*Candida*	53 (0.14)	16 (0.05)	23 (0.10)	28 (0.16)	22 (0.18)	18 (0.21)	13 (0.20)

**Abbreviations:** CoNS, Coagulase-negative staphylococci; MSSA, Methicillin-Sensitive *Staphylococcus aureus*; MRSA Methicillin-Resistant *Staphylococcus aureus*.

a If the number of organisms is 10 or less, then it is reported as ≤10.

b The denominator is the number of donors with at least one culture. Each donor might have more than one positive blood culture, and all cultures per donor are combined.

c Amp-C producing organisms included *Citrobacter freundii*, *Enterobacter cloacae*, Klebsiella aerogenes.

### Factors associated with organism positivity within 2 days of admission

A total of 77,164 donors had at least one blood culture within 2 days of admission. Among these donors, 28,505 (37%) had 1 culture in that period, 36,663 (48%) had 2, and 6259 (8%) had 3. Overall, 8130 donors (10.5%) had at least one positive blood culture within 2 days of admission, and among them, 6965 (86%) had one organism identified, 897 (11%) had two, and 268 (3%) had 3 or more. Compared with White donors, a higher percentage of Black donors had positive blood cultures, whereas Asian donors had a lower percentage. A higher percentage of donors dying of cardiovascular events, drug intoxication, and other natural causes had positive blood cultures. Donors with a positive blood culture had a higher SDI score (indicating higher deprivation) than those with a negative blood culture (median 62 vs. 57) (Table 3).

**Table 3:** Characteristics of donors with blood culture results within 2 days of admission.^a^

Variable	Total culture within 2 days of admission (N = 77,164)	Negative culture within 2 days of admission (N = 69,034)	Positive culture within 2 days of admission (N = 8130)	*p*-value
Donor age (median, years)	45 [33, 56]	45 [33, 56]	46 [35, 57]	<0.0001^d^
Donor age (years)				<0.0001^c^
18–39	29,793 (38.6)	26,924 (90.4)	2869 (9.6)	
40–49	15,838 (20.5)	14,098 (89.0)	1740 (11.0)	
50–59	18,026 (23.4)	15,983 (88.7)	2043 (11.3)	
≥60	13,507 (17.5)	12,029 (89.1)	1478 (10.9)	
Donor sex				0.68^c^
Female	30,572 (39.6)	27,334 (89.4)	3238 (10.6)	
Male	46,592 (60.4)	41,700 (89.5)	4892 (10.5)	
Donor race and ethnicity				<0.0001^c^
White, non-Hispanic	51,878 (67.2)	46,534 (89.7)	5344 (10.3)	
Black, non-Hispanic	10,917 (14.1)	9551 (87.5)	1366 (12.5)	
Hispanic/Latino	11,163 (14.5)	10,051 (90.0)	1112 (10.0)	
Asian, non-Hispanic	1991 (2.6)	1839 (92.4)	152 (7.6)	
Other	1105 (1.4)	961 (87.0)	144 (13.0)	
Unknown	110 (0.1)	98 (89.1)	12 (10.9)	
Mechanism of death				<0.0001^c^
Asphyxiation	3313 (4.3)	3002 (90.6)	311 (9.4)	
Blunt injury	10,267 (13.3)	9787 (95.3)	480 (4.7)	
Cardiovascular event	17,341 (22.5)	14,850 (85.6)	2491 (14.4)	
Drowning	329 (0.4)	301 (91.5)	28 (8.5)	
Drug intoxication	13,171 (17.1)	11,498 (87.3)	1673 (12.7)	
Gunshot or Stab	5301 (6.9)	5101 (96.2)	200 (3.8)	
ICH/stroke	21,183 (27.5)	19,364 (91.4)	1819 (8.6)	
Seizure	822 (1.1)	722 (87.8)	100 (12.2)	
Other natural causes	5437 (7.0)	4409 (81.1)	1028 (18.9)	
Alcohol use^b^				0.07
No	56,967 (76.5)	51,104 (89.7)	5863 (10.3)	
Yes	17,454 (23.5)	15,574 (89.2)	1880 (10.8)	
History of drug use^b^				0.0015^c^
No	35,739 (46.7)	32,122 (89.9)	3617 (10.1)	
Yes	40,756 (53.3)	36,343 (89.2)	4413 (10.8)	
Any positive of HBV/HCV/HIV				<0.0001^c^
No	68,935 (89.3)	61,852 (89.7)	7083 (10.3)	
Yes	8229 (10.7)	7182 (87.3)	1047 (12.7)	
PHS risk^b^				<0.0001^c^
No	58,581 (75.9)	52,719 (90.0)	5862 (10.0)	
Yes	18,578 (24.1)	16,311 (87.8)	2267 (12.2)	
Types of deceased donors^b^				0.0016^c^
DBD	57,703 (74.8)	51,740 (89.7)	5963 (10.3)	
DCD	19,458 (25.2)	17,291 (88.9)	2167 (11.1)	
SDI (median)^b^	58 [32, 80]	57 [32, 80]	62 [35, 84]	<0.0001^d^
SDI (quintile)^b^				<0.0001^c^
Q1 (0–19)	10,642 (14.1)	9635 (90.5)	1007 (9.5)	
Q2 (20–39)	13,398 (17.7)	12,117 (90.4)	1281 (9.6)	
Q3 (40–59)	15,225 (20.2)	13,683 (89.9)	1542 (10.1)	
Q4 (60–79)	16,591 (22.0)	14,875 (89.7)	1716 (10.3)	
Q5 (80–100)	19,654 (26.0)	17,218 (87.6)	2436 (12.4)	

**Abbreviations:** DBD, donation after brain death; DCD, donation after circulatory death; HBV, hepatitis B virus; HCV, hepatitis C virus; HIV, human immunodeficiency virus; ICH, Intracranial hemorrhage; PHS, Public Health Service; SDI, social deprivation index.

a Statistics presented as Median [P25, P75], N (row %).

b Data is not available for all donors. Missing values: alcohol use = 2743; history of drug use = 669; types of deceased organ donors = 3; PHS risk = 5; SDI = 1654.

c *p*-value Pearson's chi-square test.

d *p*-value Wilcoxon Rank Sum test.

In multivariable models (Table 4), donors who were older, male, and Black (vs. White) had increased odds of any positive blood culture and of CoNS. When compared to donor death from intracranial hemorrhage/stroke, donor death from asphyxiation had increased odds of anaerobic organisms (odds ratio [OR] 2.47, 95% confidence interval [CI]: 1.10–5.56). Donor death from drowning had increased odds of Gram-negative organisms (OR 3.40, 95% CI: 1.57–7.36) and anaerobic organisms (OR 5.78, 95% CI: 1.35–24.7). Donor death from a cardiovascular event had increased odds of all organisms, except *S. aureus*. Donor death from other natural causes had increased odds of all organisms. In contrast, donor death from blunt injury and gunshot/stab were associated with lower odds of any positive culture (OR 0.53, 95% CI: 0.48–0.59 and OR 0.42, 95% CI: 0.36–0.49, respectively).

**Table 4:** Multivariable models of factors associated with organism positivity within 2 days of admission.

Variable	Any positive	*Staphylococcus aureus*	*Streptococcus pneumoniae*	*Streptococcus*, other species	Gram-negative	Anaerobic	CoNS
Event	N = 8130	N = 726	N = 389	N = 1189	N = 825	N = 267	N = 2551
Donor age (per 10 yrs)	1.04 (1.02, 1.06)	0.89 (0.84, 0.94)	1.07 (0.99, 1.16)	1.01 (0.96, 1.05)	1.04 (0.98, 1.10)	1.08 (0.97, 1.19)	1.08 (1.05, 1.12)
Male (vs. female)	1.11 (1.05, 1.16)	1.11 (0.95, 1.29)	1.03 (0.84, 1.27)	1.19 (1.05, 1.34)	0.97 (0.84, 1.12)	0.96 (0.75, 1.23)	1.09 (1.01, 1.19)
Race (vs. White, NH)							
Black, NH	1.18 (1.10, 1.26)	0.63 (0.49, 0.82)	1.63 (1.25, 2.12)	1.34 (1.14, 1.58)	1.34 (1.10, 1.64)	2.15 (1.57, 2.95)	1.28 (1.14, 1.43)
Hispanic/Latino	0.99 (0.92, 1.06)	0.82 (0.65, 1.04)	1.02 (0.73, 1.43)	1.13 (0.94, 1.34)	1.32 (1.07, 1.61)	1.58 (1.10, 2.28)	0.99 (0.87, 1.12)
Asian, NH	0.78 (0.66, 0.93)	0.43 (0.22, 0.83)	0.25 (0.06, 1.01)	1.12 (0.76, 1.64)	1.38 (0.92, 2.09)	2.03 (0.98, 4.18)	0.81 (0.60, 1.09)
Other	1.29 (1.08, 1.54)	1.00 (0.57, 1.74)	1.97 (1.09, 3.58)	1.44 (0.94, 2.19)	0.93 (0.50, 1.75)	1.68 (0.69, 4.13)	1.48 (1.11, 1.98)
Mechanism of death (vs. ICH/stroke)							
Asphyxiation	1.14 (1.00, 1.29)	0.54 (0.36, 0.81)	0.94 (0.42, 2.09)	1.29 (0.94, 1.78)	1.08 (0.68, 1.71)	2.47 (1.10, 5.56)	1.44 (1.16, 1.78)
Blunt injury	0.53 (0.48, 0.59)	0.30 (0.21, 0.42)	0.55 (0.30, 1.00)	0.40 (0.29, 0.55)	0.96 (0.70, 1.30)	1.06 (0.52, 2.19)	0.51 (0.42, 0.62)
Cardiovascular event	1.79 (1.67, 1.91)	0.88 (0.72, 1.07)	1.95 (1.37, 2.76)	1.98 (1.68, 2.33)	2.36 (1.92, 2.90)	5.76 (3.71, 8.93)	1.94 (1.74, 2.17)
Drowning	1.05 (0.71, 1.56)	–	1.42 (0.20, 10.35)	1.86 (0.87, 3.99)	3.40 (1.57, 7.36)	5.78 (1.35, 24.7)	0.64 (0.26, 1.56)
Drug intoxication	1.49 (1.37, 1.61)	0.38 (0.29, 0.49)	0.99 (0.63, 1.54)	1.72 (1.41, 2.11)	1.90 (1.46, 2.47)	5.21 (3.14, 8.67)	1.88 (1.63, 2.16)
Gunshot or Stab	0.42 (0.36, 0.49)	0.12 (0.06, 0.23)	0.35 (0.14, 0.89)	0.32 (0.20, 0.50)	0.80 (0.51, 1.24)	0.73 (0.25, 2.15)	0.46 (0.35, 0.61)
Seizure	1.53 (1.23, 1.90)	0.58 (0.25, 1.31)	6.57 (3.47, 12.5)	1.70 (1.02, 2.85)	1.77 (0.89, 3.50)	5.64 (2.13, 14.92)	1.61 (1.12, 2.33)
Other natural causes	2.58 (2.37, 2.81)	1.94 (1.55, 2.43)	16.2 (11.8, 22.2)	2.30 (1.85, 2.85)	4.14 (3.25, 5.27)	5.93 (3.50, 10.04)	1.93 (1.65, 2.25)
Heavy alcohol use	1.04 (0.98, 1.10)	0.85 (0.71, 1.03)	1.39 (1.08, 1.79)	1.19 (1.04, 1.37)	1.11 (0.94, 1.32)	1.10 (0.82, 1.47)	1.00 (0.91, 1.11)
History of drug use	1.00 (0.95, 1.06)	1.05 (0.88, 1.25)	1.33 (1.06, 1.67)	1.06 (0.92, 1.21)	0.73 (0.62, 0.86)	0.98 (0.74, 1.31)	0.98 (0.89, 1.08)
Any positive of HBV/HCV/HIV	1.11 (1.03, 1.19)	1.49 (1.21, 1.83)	1.17 (0.86, 1.61)	1.04 (0.87, 1.25)	1.24 (1.004, 1.53)	0.78 (0.52, 1.19)	0.89 (0.78, 1.02)
PHS risk	1.17 (1.10, 1.24)	2.20 (1.84, 2.62)	1.24 (0.96, 1.60)	1.08 (0.94, 1.26)	1.33 (1.12, 1.59)	1.07 (0.78, 1.45)	0.99 (0.89, 1.09)
DCD (vs. DBD)	0.95 (0.90, 1.01)	1.29 (1.10, 1.52)	0.37 (0.28, 0.49)	0.94 (0.82, 1.07)	0.99 (0.84, 1.16)	1.07 (0.81, 1.40)	1.08 (0.99, 1.18)
SDI (per 10 units higher deprivation)	1.03 (1.02, 1.04)	1.01 (0.98, 1.04)	1.01 (0.97, 1.05)	1.01 (0.99, 1.04)	1.01 (0.99, 1.04)	1.03 (0.99, 1.08)	1.05 (1.03, 1.06)

Results displayed as Odds Ratio (95% CI).

**Abbreviations:** CoNS, Coagulase-negative staphylococci; DBD, donation after brain death; DCD, donation after circulatory death; HBV, hepatitis B virus; HCV, hepatitis C virus; HIV, human immunodeficiency virus; ICH, Intracranial hemorrhage; NH, non-Hispanic; PHS, Public Health Service; SDI, social deprivation index; yr, year.

Donors with a history of heavy alcohol use had increased odds of *S. pneumoniae* (OR 1.39, 95% CI: 1.08–1.79) and other *Streptococcus* species (OR 1.19, 95% CI: 1.04–1.37). Donors with HBV, HCV, or HIV infection had increased odds of any positive blood culture (OR 1.11, 95% CI: 1.03–1.19), *S. aureus* (OR 1.49, 95% CI: 1.21–1.83), and Gram-negative organisms (OR 1.24, 95% CI: 1.004–1.53). Donors with PHS risk had increased odds of any positivity (OR 1.17, 95% CI: 1.10–1.24), *S. aureus* (OR 2.20, 95% CI: 1.84–2.62), and Gram-negative organisms (OR 1.33, 95% CI: 1.12–1.59). Compared with DBD, DCD had increased odds of *S. aureus* (OR 1.29, 95% CI: 1.10–1.52). Donors with higher SDI scores had increased odds of any positive blood culture (OR per 10 units higher deprivation 1.03, 95% CI: 1.02–1.04) and CoNS (OR 1.05, 95% CI: 1.03–1.06).

## Discussion

In this study, using data representing 83% of deceased adult donors in the United States from 2014 to 2024, the percentage of donors with positive donor blood cultures increased over time, reaching 13.5% in 2024. This trend was observed across multiple organism categories, including Gram-positive, Gram-negative, and suspected contamination. The percentage of candidemia remained low but increased during hospitalization. A multivariable model identified donor-related factors associated with positive blood cultures within the first two days of hospital admission.

In our study, donor blood culture positivity was highest on day 1 of admission (15.2%) and declined to ∼6% from day 2 to day 7. This pattern aligns with a single-center study from Spain, where 5% (29/569) of donors had bacteremia at the time of organ procurement.^14^ These findings are consistent with the American Society of Transplantation (AST) guideline, which recommends that donors receive targeted antibiotics for at least 24–48 h, with evidence of clinical improvement, before organ procurement.^1^ The AST guideline also advises that transplant recipients receive 7–14 days of antibiotics targeting the donor's organism.^1^ Although these recommendations are primarily based on expert opinion, they have been associated with a reduced risk of DDIs. However, such practice might lead to underreporting of DDIs by decreasing the severity of infections and complicating future efforts to assess the optimal duration of antibiotic therapy in bacteremic donors.

During donor hospitalization, the increasing trends in *Candida* and Gram-negative organisms might suggest that longer hospital stays increase the risk of hospital-acquired bacteremia.^15^ On the other hand, the proportion of CoNS was highest on day 1 (4.9%) and remained stable from day 2 to day 7 (<2%), indicating that blood culture suspected contamination is most likely during urgent sampling situations (day 1).^16^ The risk of blood culture suspected contamination persists while donors remain on life support devices prior to organ procurement.

A study from the 1990s reported that 5% (95/1775) of donors in the New England area had positive blood cultures at any time before organ procurement,^17^ while our study found 7% of donors had positive blood cultures in 2014, but the percentage increased over the years. The increasing annual percentage of bacteremic donors observed in our study may be attributed to two reasons. First, since treated bacteremia in donors has generally not been shown to negatively impact recipient outcomes,^6^^,^^7^^,^^14^^,^^17^^,^^18^ the donor pool has been gradually expanded to include such cases. Second, the increased utilization of donors with HBV or HCV infection, greater use of DCD donors, and changes in donor causes of death may have contributed to the rising proportion of donors with positive blood cultures over time. For example, between 2014 and 2024, the proportion of donors whose cause of death was drug intoxication increased from 7.3% to 12.2%, whereas deaths due to gunshot wounds and blunt injuries decreased from 8.2% to 5.5% and from 21.3% to 14.3%, respectively.^19^ In addition, the use of DCD donors increased substantially, from 2% in 2000 to nearly 49% in 2025.^20^ The use of HCV-positive donors has increased substantially in the direct-acting antiviral era,^21^ whereas the availability of effective antiviral prophylaxis has facilitated broader acceptance of HBV-positive donors.^22^ Notably, implementation of the 2020 U.S. Public Health Service (PHS) guideline was not associated with reduced donor utilization rates.^23^

In our analysis, donors with a history of drug use or with HIV/HBV/HCV infection had increased odds of *S. aureus* bacteremia, consistent with the elevated risk of *S. aureus* colonization/infection in these populations.^24^, ^25^, ^26^, ^27^ In contrast, death from drug intoxication alone (compared to death from intracranial hemorrhage/stroke) was associated with decreased odds of *S. aureus* bacteremia, possibly reflecting that *S. aureus* infection was not the proximate cause of death in these donors. The higher odds of *S. aureus* bacteremia among DCD donors may reflect differences in donor characteristics or duration of hospitalization (hospital-acquired infection). Donors who died from asphyxiation or seizure had increased odds of anaerobic bacteremia, likely due to translocation of oral flora into the bloodstream. The higher odds of Gram-negative organisms among drowning donors suggest that water may serve as a source of *Enterobacterales*. Donors with a history of heavy alcohol use had greater odds of *Streptococcus* infection, consistent with alcohol-related dysregulation of the immune system.^28^ Donors having PHS risk were originally identified as having an increased risk of HIV/HBV/HCV transmission to recipients.^29^ In our study, PHS risk was also associated with donor bacteremia. Finally, older, male, and Black donors were more likely to have any positive blood culture, consistent with existing literature suggesting these populations are more vulnerable to bacteremia.^30^, ^31^, ^32^ The observed association with race should be interpreted with caution. These findings can reflect unmeasured structural and social determinants of health, including differences in healthcare access, socioeconomic conditions, prevalence of underlying comorbidities, patterns of healthcare utilization, and other systemic factors that are not captured in the OPTN database. Low socioeconomic status (higher SDI score) is also a known risk factor for bacteremia.^33^

A U.S.-based survey of infectious disease providers found that acceptance rates for bacteremic donors varied by causative organism.^34^ Notably, the highest acceptance rate (76.3%) was reported for donors with *Streptococcus* bacteremia.^34^ In our study, *Streptococcus* species were identified as the second most common organisms, following CoNS. Even before our epidemiological data became available, experienced infectious disease providers were likely frequently consulted for cases involving *Streptococcus*, which may have contributed to their confidence in managing donors with *Streptococcus* bacteremia.

This study has several limitations. First, reporting bias exists because not all donors in the database had blood culture data. Sampling bias exists because blood culture testing practices varied during hospitalization. Second, ∼40% of the Gram-positive and Gram-negative cultures lacked finalized organism identification, and about half of the *S. aureus* isolates could not be classified as MSSA or MRSA. Third, all *Streptococcus* species were categorized as Gram-positive, although 65% were not *S. agalactiae, S. pneumoniae*, or *S. pyogenes*. Due to the lack of detailed donor clinical history and ongoing changes in *Streptococcus* taxonomy, some of these isolates may represent contamination. Likewise, some CoNS isolated may represent true infection, depending on the clinical context. Fourth, the cause of death classification in the UNOS may not accurately reflect the donor's true cause of death, which is often multifactorial. Fifth, because this study focused solely on blood culture results, potential interactions between blood cultures and other specimen types (e.g., urine, cerebrospinal fluid, or sputum cultures) were not evaluated. These interactions may also influence the donor's cause of death. Sixth, organism susceptibility data, particularly important for *Enterobacterales*, were not available in this database, as such information is typically stored as PDF files on the UNOS platform and not captured in analyzable format. Seventh, the number of community-acquired bacteremia cases was relatively low (∼8000 over the past decade), and only a limited number of organisms had sufficient sample sizes for robust modeling. Eighth, detailed PHS risk variables have been collected in the UNOS database since 2023. Therefore, in this study, we could only report PHS risk as a dichotomous Yes/No variable and were unable to analyze each PHS risk factor. Abovementioned limitations restricted the scope and power of analyses.

To our knowledge, this study presents the first national-level epidemiological data on donor blood cultures. Our findings highlight that the expansion of the donor pool (donors with PHS risk, donors with HBV/HCV infection, and the opioid epidemic) has been accompanied by an increased percentage of bacteremic donors. However, this risk must be weighed against the reality that life-saving organs are sometimes transplanted before finalized donor blood culture results become available, with some returning positive only hours or days after transplantation. Future studies should focus on optimizing peri-transplant antimicrobial prophylaxis based on donor factors to prevent DDIs, as well as evaluating the impact of specific organisms on allograft outcomes.

## Contributors

C-YC (Conception, Writing—original draft, Writing—review & editing), SA (Conception, Data curation, Formal analysis, Writing—original draft, Writing—review & editing), RL (Supervision, Writing—review & editing), ES and WA (Writing—review & editing), JDS (Conception, Supervision, Writing—review & editing). All authors had access to all the data. C-YC, SA, RL, and JDS validated the results. The corresponding authors have final responsibility for the decision to submit for publication.

## Data sharing statement

The de-identified datasets generated during and/or analyzed during the current study are available from the corresponding author on reasonable request.

## Declaration of interests

The data reported here have been supplied by UNOS as the contractor for the Organ Procurement and Transplantation Network (OPTN). The interpretation and reporting of these data are the responsibility of the author(s) and in no way should be seen as an official policy of or interpretation by the OPTN or the U.S. Government. Rocio Lopez received grants from the One Legacy Foundation and the Gift of Life Foundation and is a member of a technical expert panel sponsored by the Association for Organ Procurement Organizations. Jesse D. Schold received grants from Kamada Pharmaceuticals, NIH, Department of Defense, One Legacy Foundation, and Donor Alliance Foundation. He consults for Sanofi Inc., eGenesis Inc., and Verici Dx. He is the Chair of the Data Advisory Committee at UNOS, a member of a technical expert panel sponsored by the Association for Organ Procurement Organizations. The remaining authors have no conflicts to report.
