# Supplementary material for: Epidemiology of adult deceased solid organ donors with positive blood cultures in the USA: a retrospective registry-based study
Source: Lancet Reg Health Am. 2026 Sep 12;64:101638. doi: 10.1016/j.lana.2026.101638 (PMC13586872; doi:10.1016/j.lana.2026.101638)
Supplement: Supplementary file 1 [file mmc1.pdf]

Epidemiology of adult deceased solid organ donors with positive blood cultures in the USA: a retrospective registry-based study

Table of Contents

Supplementary methods 2

Table S1. Organism classification in this study. 4

Table S2. Blood culture data available for recovered deceased donors, stratified by year (2014 – 2024).[a] 6

Table S3. Validation of organism coding accuracy by manual review. 7

Table S4. Characteristics of donors with positive and negative blood culture results. ... 8

Table S5. Incidence rate ratios comparing donor positivity from donor blood culture results over the years. 11

Table S6. Detailed incidence of donor positivity from donor blood culture results, list of organisms to supplement Table 1, stratified by year (2014 – 2024). 12

Table S7. Detailed donor blood culture results, list of organisms to supplement Table 1, stratified by day of admission (days 1–7). 15

Figure S1. Inclusion flow chart. 17

Figure S2. Number and percentage of donors having blood culture results from admission day 1 to day 7. 18

Figure S3. Total Number of blood culture and percentage of positive blood culture results from admission day 1 to day 7. 19

Supplementary references 20

Supplementary methods

Statistical analysis

We graphed the total number of cultures available by donation year and the number and percent of cultures that were negative and positive. For donors included in the study, we graphed the number still admitted to the hospital from day 1 (admission day) to day 7, and the number and percentage with a culture each day.

We summarized the results of our organism validation by reporting the true positive, true negative, false positive, false negative, sensitivity, specificity, positive predictive value, negative predictive value, and accuracy for each organism selected.

We summarized the percentage of donors with positive blood cultures and the percentage of each organism overall and by year of donation. We graphed the incidence of gram-positive, gram-negative, anaerobic, suspected contamination, and *Candida* among donors with available culture by year and used Poisson models to evaluate changes in incidence over time. Then we also graphed the following: (1) percentage of donors with presence of specific organisms out of those with a Gram-positive culture by year, (2) percentage of donors with presence of specific organisms out of those with a Gram-negative culture by year, (3) percentage of donors with presence of specific organisms out of those with suspected contamination in the culture by year. We also summarized the percentage of donors with positive organisms each day within the donor admission.

The clinical and research activities being reported are consistent with the Principles of the Declaration of Istanbul as outlined in the 'Declaration of Istanbul on Organ Trafficking and Transplant Tourism.

Donor with Hepatitis B

History of HBV in this study was defined as a positive HBV nucleic acid test (NAT),

positive hepatitis B core antibody, or positive hepatitis B surface antigen.

Public Health Service (PHS risk)

In July 2013, the U.S. Public Health Service (PHS) published guidelines for reducing the transmission of human immunodeficiency virus (HIV), hepatitis B virus (HBV), and hepatitis C virus (HCV) through organ transplantation [1]. In this study, we used PHS risk as a variable to assess donor risk of bacteremia within 2 days of admission. Although PHS risk comprised multiple individual items, the SRTR database summarized it as an overall Yes/No variable.

Social Deprivation Index (SDI)

We used the 2019 Social Deprivation Index (SDI) developed by the Robert Graham Center [2]. This 2019 index is calculated using the 2015-2019 American Community Survey and is a composite of the following seven demographic characteristics: (1) percent population less than 100% federal poverty level (FPL), (2) percent population 25 years or more with less than 12 years of education, (3) percent non-employed for population 16-64 years, (4) percent households living in renter-occupied housing units, (5) percent households living in crowded housing units, (6) percent single parent families with dependents < 18 years, and (7) percent households with no vehicle. The SDI was obtained at the Zip Code Tabulation Area level, and a Zip Code Tabulation Area code crosswalk was used to merge with SRTR data at the zip code level. The SDI score ranges from 0 (least deprived) to 100 (most deprived); we divided it into 5 groups: Quintile 1: 0-19, Quintile 2: 20-39, Quintile 3: 40-59, Quintile 4: 60-79, and Quintile 5: 80-100.

Race

The UNOS race variable available in the data has the following race categories: Non-Hispanic White, Non-Hispanic Black, Hispanic/Latino, Non-Hispanic Asian, Non-Hispanic Amer Ind/Alaska Native, Non-Hispanic Native Hawaiian/other Pacific Islander, Non-Hispanic Multiracial. The racial/ethnic categories with the lowest counts were combined into a category of “Other”

Table S1. Organism classification in this study.

Gram-positive organism	➢ Gram-positive with no final report<br>➢ MSSA (Methicillin-Sensitive *Staphylococcus aureus*)<br>➢ MRSA (Methicillin-Resistant *Staphylococcus aureus*)<br>➢ *Staphylococcus aureus*, non-specific<br>➢ *Staphylococcus lugdunensis*<br>➢ *Streptococcus agalactiae*<br>➢ *Streptococcus pyogenes*<br>➢ *Streptococcus pneumoniae*<br>➢ *Streptococcus*, other species<br>➢ *Enterococcus faecalis*<br>➢ *Enterococcus faecium*<br>➢ *Enterococcus*, other species
Gram-negative organism	➢ Gram-negative with no final report<br>➢ *Enterobacterales* (AmpC-producing bacteria)<br>■ *Citrobacter freundii*<br>■ *Enterobacter cloacae*<br>■ *Klebsiella aerogenes*<br>➢ Enterobacterales (not AmpC-producing bacteria)<br>■ *Citrobacter* non-*freundii*<br>■ *Enterobacter* non-*cloacae*<br>■ *Klebsiella* non-*aerogenes*<br>■ *Escherichia coli*<br>■ *Haemophilus influenzae*<br>■ *Salmonella*<br>■ *Serratia*<br>■ *Shigella*<br>■ *Proteus*<br>➢ *Acinetobacter baumanii*<br>➢ *Acinetobacter*, other species<br>➢ *Pseudomonas aeruginosa*<br>➢ *Stenotrophomonas maltophilia*
Anaerobic	➢ *Actinomyces*<br>➢ *Anaerococcus*<br>➢ *Bacteroides*<br>➢ *Clostridia / Clostridioides*<br>➢ *Eikenella*<br>➢ *Finegoldia*<br>➢ *Fusobacterium*<br>➢ *Kingella Kingae*<br>➢ *Lactobacillus*<br>➢ *Parvimonas*<br>➢ *Pasteurella*<br>➢ *Peptococcus*<br>➢ *Peptostreptococcus*<br>➢ *Porphyromonas*<br>➢ *Prevotella*
Suspected contamination	➢ *Aerococcus*<br>➢ *Bacillus*, non-*anthracis*<br>➢ Coagulase-negative staphylococci

	➢ *Corynebacterium* (Diphtheroid)<br>➢ *Micrococcus*<br>➢ *Propionibacterium* (*Cutibacterium*)
Candida	➢ *Candida albicans*<br>➢ *Candida auris*<br>➢ *Candida dubliniensis*<br>➢ *Candida glabrata*<br>➢ *Candida krusei*<br>➢ *Candida lusitaniae*<br>➢ *Candida parapsilosis*<br>➢ *Candida tropicalis*

Table S2. Blood culture data available for recovered deceased donors, stratified by year (2014 – 2024).[a]

Factor	Total (N=123,625)	2014 (N=7,611)	2015 (N=8,075)	2016 (N=8,954)	2017 (N=9,311)	2018 (N=9,760)	2019 (N=10,845)	2020 (N=11,626)	2021 (N=12,703)	2022 (N=13,713)	2023 (N=15,226)	2024 (N=15,801)
**Days Admit to Donation**	5 [4, 8]	4 [3, 6]	5 [4, 6]	5 [4, 7]	5 [4, 7]	5 [4, 7]	5 [4, 7]	5 [4, 7]	6 [4, 8]	6 [5, 8]	6 [5, 9]	6 [5, 9]
**Had at least 1 blood culture**	102,485 (83)	5,472 (72)	6,131 (76)	7,041 (79)	7,552 (81)	8,043 (82)	9,107 (84)	9,702 (83)	10,804 (85)	11,642 (85)	13,110 (86)	13,881 (88)
**Had Culture within 2 days of Admit [a]**	77,164 (75)	4,196 (77)	4,645 (76)	5,379 (76)	5,781 (77)	6,161 (77)	6,933 (76)	7,417 (76)	8,020 (74)	8,689 (75)	9,685 (74)	10,258 (74)
**Had Culture more than 2 days after Admit [a]**	50,111 (49)	2,110 (39)	2,472 (40)	2,929 (42)	3,223 (43)	3,525 (44)	4,167 (46)	4,736 (49)	5,751 (53)	6,282 (54)	7,141 (54)	7,775 (56)

a. Statistics presented as Median [P25, P75], N (column %).

b. Percentage for culture within 2 days and more than 2 days after admission calculated among those that have at least 1 culture.

Table S3. Validation of organism coding accuracy by manual review.[a]

Organism	TP	TN	FP	FN	Sensitivity	Specificity	PPV	NPV	Accuracy
**Gram positive**	120	147	18	5	96.0 (92.6, 99.4)	89.1 (84.3, 93.8)	87.0 (81.3, 92.6)	96.7 (93.9, 99.5)	92.1 (89.0, 95.2)
Staphylococcus	163	417	9	3	98.2 (96.2, 100)	97.9 (96.5, 99.3)	94.8 (91.4, 98.1)	99.3 (98.5, 100)	98.0 (96.8, 99.1)
S. aureus	59	524	9	0	100 (100, 100)	98.3 (97.2, 99.4)	86.8 (78.7, 94.8)	100 (100, 100)	98.5 (97.5, 99.5)
S. epidermidis	69	512	6	5	93.2 (87.5, 99.0)	98.8 (97.9, 99.8)	92.0 (85.9, 98.1)	99.0 (98.2, 99.9)	98.1 (97.1, 99.2)
S. lugdunensis	34	248	8	0	100 (100, 100)	96.9 (94.7, 99.0)	81.0 (69.1, 92.8)	100 (100, 100)	97.2 (95.4, 99.1)
Streptococcus	105	472	14	1	99.1 (97.2, 100)	97.1 (95.6, 98.6)	88.2 (82.4, 94.0)	99.8 (99.4, 100)	97.5 (96.2, 98.7)
S. pneumoniae	62	520	8	2	96.9 (92.6, 100)	98.5 (97.4, 99.5)	88.6 (81.1, 96.0)	99.6 (99.1, 100)	98.3 (97.3, 99.3)
Enterococcus	47	237	6	0	100 (100, 100)	97.5 (95.6, 99.5)	88.7 (80.1, 97.2)	100 (100, 100)	97.9 (96.3, 99.6)
**Gram negative**	153	417	21	1	99.4 (98.1, 100)	95.2 (93.2, 97.2)	87.9 (83.1, 92.8)	99.8 (99.3, 100)	96.3 (94.8, 97.8)
Escherichia	65	524	3	0	100 (100, 100)	99.4 (98.8, 100)	95.6 (90.7, 100)	100 (100, 100)	99.5 (98.9, 100.0)
Klebsiella	51	532	9	0	100 (100, 100)	98.3 (97.3, 99.4)	85.0 (76.0, 94.0)	100 (100, 100)	98.5 (97.5, 99.5)
**Anaerobic**	46	235	7	2	95.8 (90.2, 100)	97.1 (95.0, 99.2)	86.8 (77.7, 95.9)	99.2 (98.0, 100)	96.9 (94.9, 98.9)
**Suspected contamination**	109	162	15	4	96.5 (93.1, 99.9)	91.5 (87.4, 95.6)	87.9 (82.2, 93.6)	97.6 (95.3, 99.9)	93.4 (90.6, 96.3)
Bacillus*, non-*anthracis	45	236	9	0	100 (100, 100)	96.3 (94.0, 98.7)	83.3 (73.4, 93.3)	100 (100, 100)	96.9 (94.9, 98.9)
Candida	34	541	17	0	100 (100, 100)	97.0 (95.5, 98.4)	66.7 (53.7, 79.6)	100 (100, 100)	97.1 (95.8, 98.5)

A total of 882 random blood draw samples were manually reviewed for validation.

Abbreviations: FN, false negative; FP, false positive; PPV, positive predictive value; NPV, negative predictive value; TN, true negative; TP, true positive.

a. Wald 95% confidence intervals reported.

Table S4. Characteristics of donors with positive and negative blood culture results.[a]

Variable	Total (N=102,485)	Negative (N=91,077)	Positive (N=11,408)	p-value
**Donor age (years)**	45 [33, 56]	45 [33, 56]	46 [34, 56]	<0.0001[d]
**Donor age (years)**				<0.0001[c]
18 - 39	39,620 (38.7)	35,478 (89.5)	4,142 (10.5)	
40 - 49	21,099 (20.6)	18,648 (88.4)	2,451 (11.6)	
50 - 59	24,043 (23.5)	21,205 (88.2)	2,838 (11.8)	
≥60	17,723 (17.3)	15,746 (88.8)	1,977 (11.2)	
**Donor sex**				0.07[c]
Female	39,962 (39.0)	35,602 (89.1)	4,360 (10.9)	
Male	62,523 (61.0)	55,475 (88.7)	7,048 (11.3)	
**Donor race and ethnicity**				<0.0001[c]
White, Non-Hispanic	67,016 (65.4)	59,726 (89.1)	7,290 (10.9)	
Black, Non-Hispanic	15,163 (14.8)	13,282 (87.6)	1,881 (12.4)	
Hispanic/Latino	15,879 (15.5)	14,094 (88.8)	1,785 (11.2)	
Asian, Non-Hispanic	2,786 (2.7)	2,550 (91.5)	236 (8.5)	
Other	1,495 (1.5)	1,297 (86.8)	198 (13.2)	
Unknown	146 (0.1)	128 (87.7)	18 (12.3)	
**Mechanism of death**				<0.0001[c]
Asphyxiation	4,635 (4.5)	4,144 (89.4)	491 (10.6)	
Blunt injury	16,401 (16.0)	15,282 (93.2)	1,119 (6.8)	
Cardiovascular event	21,683 (21.2)	18,551 (85.6)	3,132 (14.4)	
Drowning	452 (0.4)	411 (90.9)	41 (9.1)	
Drug intoxication	15,699 (15.3)	13,594 (86.6)	2,105 (13.4)	
Gunshot or stab	6,649 (6.5)	6,345 (95.4)	304 (4.6)	
Seizure	1,049 (1.0)	910 (86.7)	139 (13.3)	
ICH/stroke	28,637 (27.9)	25,967 (90.7)	2,670 (9.3)	
Other natural causes	7,280 (7.1)	5,873 (80.7)	1,407 (19.3)	
**Heavy alcohol use**[b]				0.0005[c]

Variable	Total (N=102,485)	Negative (N=91,077)	Positive (N=11,408)	p-value
No	75,845 (76.8)	67,644 (89.2)	8,201 (10.8)	
Yes	22,950 (23.2)	20,280 (88.4)	2,670 (11.6)	
**History of drug use** [b]				0.0003[c]
No	48,539 (47.8)	43,332 (89.3)	5,207 (10.7)	
Yes	52,989 (52.2)	46,925 (88.6)	6,064 (11.4)	
**Any positive of HBV/HCV/HIV**				<0.0001[c]
No	91,822 (89.6)	81,820 (89.1)	10,002 (10.9)	
Yes	10,663 (10.4)	9,257 (86.8)	1,406 (13.2)	
**PHS risk** [b]				<0.0001[c]
No	78,907 (77.0)	70,490 (89.3)	8,417 (10.7)	
Yes	23,568 (23.0)	20,578 (87.3)	2,990 (12.7)	
**Types of deceased donors** [b]				<0.0001[c]
DBD	74,319 (72.5)	66,372 (89.3)	7,947 (10.7)	
DCD	28,163 (27.5)	24,702 (87.7)	3,461 (12.3)	
**SDI (median)**	58 [33, 81]	58 [32, 80]	62 [36, 84]	<0.0001[d]
**SDI (quintiles)**				<0.0001[c]
Q1	13,630 (13.6)	12,285 (90.1)	1,345 (9.9)	
Q2	17,539 (17.5)	15,753 (89.8)	1,786 (10.2)	
Q3	20,206 (20.2)	18,030 (89.2)	2,176 (10.8)	
Q4	22,140 (22.1)	19,667 (88.8)	2,473 (11.2)	
Q5	26,551 (26.5)	23,172 (87.3)	3,379 (12.7)	

Abbreviations: DBD, donation after brain death; DCD, donation after circulatory death; HBV, hepatitis B virus; HCV, hepatitis C virus; HIV, human immunodeficiency virus; ICH, Intracranial hemorrhage; PHS, Public Health Service; SDI, social deprivation index.

a. Statistics presented as Median [P25, P75], N (row %).
b. Data is not available for all donors. Missing values: alcohol use = 3,690; history of drug use = 957; types of deceased organ donors = 3; PHS risk = 10; SDI = 2,419.

c. p-value Pearson's chi-square test.
d. p-value Wilcoxon Rank Sum test.

Table S5. Incidence rate ratios comparing donor positivity from donor blood culture results over the years.

Year	Any Positive Culture IRR (95% CI)	Gram-positive IRR (95% CI)	Gram-negative IRR (95% CI)	Contamination IRR (95% CI)	Anaerobic IRR (95% CI)	*Candida* IRR (95% CI)
**2014**	Ref	Ref	Ref	Ref		
**2015**	1.12 (0.98, 1.28)	1.05 (0.87, 1.27)	1.30 (0.89, 1.89)	1.19 (0.97, 1.46)		
**2016**	1.23 (1.08, 1.40)	1.20 (1.01, 1.43)	1.23 (0.85, 1.78)	1.19 (0.97, 1.45)		
**2017**	1.19 (1.04, 1.35)	1.27 (1.07, 1.51)	1.06 (0.73, 1.54)	1.07 (0.88, 1.31)	Ref	
**2018**	1.38 (1.22, 1.56)	1.33 (1.12, 1.57)	1.72 (1.22, 2.4)	1.36 (1.12, 1.64)	1.41 (0.72, 2.8)	Ref
**2019**	1.49 (1.32, 1.67)	1.51 (1.28, 1.77)	1.41 (1.00, 1.99)	1.42 (1.18, 1.71)	1.54 (0.80, 2.9)	1.18 (0.56, 2.5)
**2020**	1.62 (1.44, 1.82)	1.57 (1.34, 1.85)	1.59 (1.14, 2.2)	1.73 (1.45, 2.1)	1.89 (1.01, 3.5)	1.38 (0.68, 2.8)
**2021**	1.75 (1.56, 1.96)	1.57 (1.34, 1.84)	1.56 (1.12, 2.2)	2.0 (1.68, 2.4)	2.3 (1.29, 4.3)	1.55 (0.78, 3.1)
**2022**	1.93 (1.73, 2.2)	1.76 (1.51, 2.0)	2.1 (1.49, 2.8)	2.2 (1.84, 2.6)	1.76 (0.95, 3.2)	2.1 (1.11, 4.1)
**2023**	1.88 (1.68, 2.1)	1.66 (1.43, 1.94)	1.81 (1.32, 2.5)	2.2 (1.85, 2.6)	1.89 (1.04, 3.4)	1.79 (0.93, 3.4)
**2024**	1.93 (1.73, 2.1)	1.67 (1.44, 1.94)	1.94 (1.41, 2.7)	2.3 (1.91, 2.7)	2.4 (1.33, 4.2)	2.1 (1.12, 4.0)

Overall, p-value for year <0.0001 for positive culture, Gram-positive, Gram-negative, and suspected contamination.

Overall, p-value=0.033 for year 2017-2024 for anaerobic.

Overall, p-value=0.08 for year 2018-2024 for *Candida*.

Table S6. Detailed incidence of donor positivity from donor blood culture results, list of organisms to supplement Table 1, stratified by year (2014 – 2024).[ab]

organism	Total (N=102,485)	2014 (N=5,472)	2015 (N=6,131)	2016 (N=7,041)	2017 (N=7,552)	2018 (N=8,043)	2019 (N=9,107)	2020 (N=9,702)	2021 (N=10,804)	2022 (N=11,642)	2023 (N=13,110)	2024 (N=13,881)
**Gram-positive**												
Staphylococcus lugdunensis	42 (0.04)	<10	<10	<10	<10	<10	<10	<10	<10	<10	<10	<10
Enterococcus, other species	77 (0.08)	≤10	≤10	≤10	≤10	≤10	≤10	≤10	≤10	≤10	12 (0.09)	≤10
**Gram-negative, Amp-C producing**												
Citrobacter freundii	≤10	≤10	≤10	≤10	≤10	≤10	≤10	≤10	≤10	≤10	≤10	≤10
Enterobacter cloacae	64 (0.06)	≤10	≤10	≤10	≤10	≤10	≤10	≤10	≤10	≤10	11 (0.08)	11 (0.08)
Klebsiella aerogenes	39 (0.04)	≤10	≤10	≤10	≤10	≤10	≤10	≤10	≤10	≤10	≤10	≤10
**Gram-negative, Not AmpC producing**												
Citrobacter non-*freundii*	11 (0.01)	≤10	≤10	≤10	≤10	≤10	≤10	≤10	≤10	≤10	≤10	≤10
Enterobacter non-*cloacae*	38 (0.04)	≤10	≤10	≤10	≤10	≤10	≤10	≤10	≤10	≤10	≤10	≤10
Klebsiella non-*aerogenes*	221 (0.22)	≤10	≤10	≤10	≤10	≤10	23 (0.25)	24 (0.25)	21 (0.19)	43 (0.37)	28 (0.21)	47 (0.34)
Escherichia coli	223 (0.22)	≤10	12 (0.20)	≤10	≤10	19 (0.24)	12 (0.13)	27 (0.28)	19 (0.18)	26 (0.22)	41 (0.31)	47 (0.34)
Haemophilus influenzae	66 (0.06)	≤10	≤10	≤10	≤10	≤10	≤10	≤10	≤10	≤10	≤10	13 (0.09)
Salmonella	≤10	≤10	≤10	≤10	≤10	≤10	≤10	≤10	≤10	≤10	≤10	≤10
Serratia	83 (0.08)	≤10	≤10	≤10	≤10	≤10	≤10	≤10	15 (0.14)	13 (0.11)	≤10	13 (0.09)

organism	Total (N=102,485)	2014 (N=5,472)	2015 (N=6,131)	2016 (N=7,041)	2017 (N=7,552)	2018 (N=8,043)	2019 (N=9,107)	2020 (N=9,702)	2021 (N=10,804)	2022 (N=11,642)	2023 (N=13,110)	2024 (N=13,881)
Shigella	≤10	≤10	≤10	≤10	≤10	≤10	≤10	≤10	≤10	≤10	≤10	≤10
Proteus	30 (0.03)	≤10	≤10	≤10	≤10	≤10	≤10	≤10	≤10	≤10	≤10	≤10
**Other gram-negative**												
Acinetobacter baumanii	56 (0.05)	≤10	≤10	≤10	≤10	≤10	≤10	≤10	≤10	≤10	≤10	≤10
Acinetobacter, other species	35 (0.03)	≤10	≤10	≤10	≤10	≤10	≤10	≤10	≤10	≤10	≤10	≤10
Stenotrophomonas maltophilia	11 (0.01)	≤10	≤10	≤10	≤10	≤10	≤10	≤10	≤10	≤10	≤10	≤10
**Suspected contamination**												
Aerococcus	31 (0.03)	≤10	≤10	≤10	≤10	≤10	≤10	≤10	≤10	≤10	≤10	≤10
Bacillus, non-anthracis	296 (0.29)	11 (0.20)	12 (0.20)	19 (0.27)	16 (0.21)	21 (0.26)	26 (0.29)	26 (0.27)	40 (0.37)	39 (0.33)	45 (0.34)	41 (0.30)
Corynebacterium	196 (0.19)	≤10	≤10	≤10	11 (0.15)	≤10	15 (0.16)	16 (0.16)	22 (0.20)	26 (0.22)	27 (0.21)	48 (0.35)
Propionibacterium	134 (0.13)	≤10	≤10	≤10	≤10	15 (0.19)	17 (0.19)	11 (0.11)	13 (0.12)	17 (0.15)	17 (0.13)	21 (0.15)
Micrococcus	159 (0.16)	≤10	≤10	11 (0.16)	≤10	≤10	≤10	14 (0.14)	18 (0.17)	27 (0.23)	21 (0.16)	33 (0.24)
**Candida**												
Candida albicans	42 (0.04)	≤10	≤10	≤10	≤10	≤10	≤10	≤10	≤10	≤10	≤10	≤10
Candida auris	≤10	≤10	≤10	≤10	≤10	≤10	≤10	≤10	≤10	≤10	≤10	≤10
Candida dubliniensis	16 (0.02)	≤10	≤10	≤10	≤10	≤10	≤10	≤10	≤10	≤10	≤10	≤10
Candida glabrata	49 (0.05)	≤10	≤10	≤10	≤10	≤10	≤10	≤10	≤10	≤10	≤10	11 (0.08)
Candida krusei	≤10	≤10	≤10	≤10	≤10	≤10	≤10	≤10	≤10	≤10	≤10	≤10
Candida lusitaniae	≤10	≤10	≤10	≤10	≤10	≤10	≤10	≤10	≤10	≤10	≤10	≤10
Candida parapsilosis	14 (0.01)	≤10	≤10	≤10	≤10	≤10	≤10	≤10	≤10	≤10	≤10	≤10
Candida tropicalis	≤10	≤10	≤10	≤10	≤10	≤10	≤10	≤10	≤10	≤10	≤10	≤10

a. If the number of organisms is 10 or less, report as ≤10.
b. The denominator is the number of donors with at least one culture. Each donor might have more than one positive blood culture and all cultures per donor are combined.

Table S7. Detailed donor blood culture results, list of organisms to supplement Table 1, stratified by day of admission (days 1–7).[ab]

Organism	1 (N=37,578)	2 (N=31,795)	3 (N=22,968)	4 (N=17,603)	5 (N=12,448)	6 (N=8,706)	7 (N=6,367)
**Gram-positive**							
Staphylococcus lugdunensis	31 (0.08)	≤10	≤10	≤10	≤10	≤10	≤10
Enterococcus, other species	31 (0.08)	16 (0.05)	≤10	≤10	≤10	≤10	≤10
**Gram-negative (AmpC-producing)**							
Citrobacter freundii	≤10	≤10	≤10	≤10	≤10	≤10	≤10
Enterobacter cloacae	17 (0.05)	≤10	≤10	≤10	≤10	≤10	≤10
Klebsiella aerogenes	≤10	≤10	≤10	≤10	≤10	≤10	≤10
**Gram-negative (not AmpC-producing)**	283 (0.75)	61 (0.19)	59 (0.26)	57 (0.32)	35 (0.28)	33 (0.38)	24 (0.38)
Citrobacter non-*freundii*	≤10	≤10	≤10	≤10	≤10	≤10	≤10
Enterobacter non-*cloacae*	16 (0.04)	≤10	≤10	≤10	≤10	≤10	≤10
Klebsiella non-*aerogenes*	79 (0.21)	19 (0.06)	20 (0.09)	24 (0.14)	13 (0.10)	15 (0.17)	12 (0.19)
Escherichia coli	123 (0.33)	30 (0.09)	23 (0.10)	16 (0.09)	≤10	≤10	≤10
Haemophilus influenzae	40 (0.11)	≤10	≤10	≤10	≤10	≤10	≤10
Salmonella	≤10	≤10	≤10	≤10	≤10	≤10	≤10
Serratia	27 (0.07)	≤10	≤10	≤10	≤10	≤10	≤10
Shigella	≤10	≤10	≤10	≤10	≤10	≤10	≤10
Proteus	17 (0.05)	≤10	≤10	≤10	≤10	≤10	≤10
**Other gram-negative**							
Acinetobacter baumanii	17 (0.05)	≤10	≤10	≤10	≤10	≤10	≤10
Acinetobacter, other species	22 (0.06)	≤10	≤10	≤10	≤10	≤10	≤10
Stenotrophomonas maltophilia	≤10	≤10	≤10	≤10	≤10	≤10	≤10
**Suspected contamination**							
Aerococcus	27 (0.07)	≤10	≤10	≤10	≤10	≤10	≤10
Bacillus, non-*anthracis*	142 (0.38)	41 (0.13)	33 (0.14)	29 (0.16)	15 (0.12)	12 (0.14)	5 (0.08)
Corynebacterium	153 (0.41)	21 (0.07)	≤10	≤10	≤10	≤10	≤10
Propionibacterium	90 (0.24)	23 (0.07)	≤10	≤10	≤10	≤10	≤10

Organism	1 (N=37,578)	2 (N=31,795)	3 (N=22,968)	4 (N=17,603)	5 (N=12,448)	6 (N=8,706)	7 (N=6,367)
Micrococcus	113 (0.30)	20 (0.06)	≤10	≤10	≤10	≤10	≤10
**Candida**							
Candida albicans	≤10	≤10	≤10	≤10	≤10	≤10	≤10
Candida auris	≤10	≤10	≤10	≤10	≤10	≤10	≤10
Candida dubliniensis	≤10	≤10	≤10	≤10	≤10	≤10	≤10
Candida glabrata	≤10	≤10	≤10	≤10	≤10	≤10	≤10
Candida krusei	≤10	≤10	≤10	≤10	≤10	≤10	≤10
Candida lusitaniae	≤10	≤10	≤10	≤10	≤10	≤10	≤10
Candida parapsilosis	≤10	≤10	≤10	≤10	≤10	≤10	≤10
Candida tropicalis	≤10	≤10	≤10	≤10	≤10	≤10	≤10

a. If the number of organisms is 10 or less, report as ≤10.

b. The denominator is the number of donors with at least one culture. Each donor might have more than one positive blood culture and all cultures per donor are combined.

Figure S1. Inclusion flow chart.

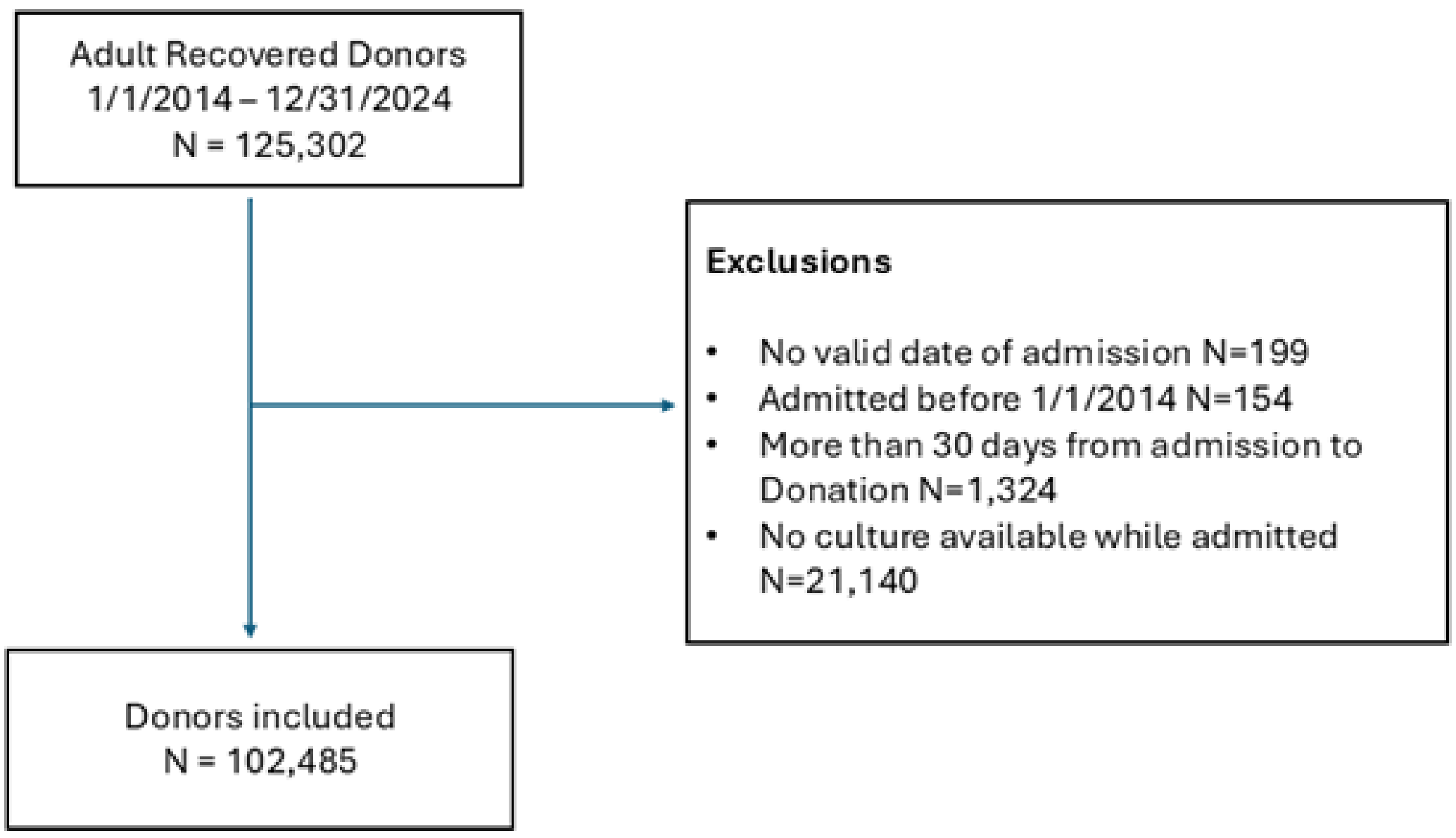

Figure S2. Number and percentage of donors having blood culture results from admission day 1 to day 7.

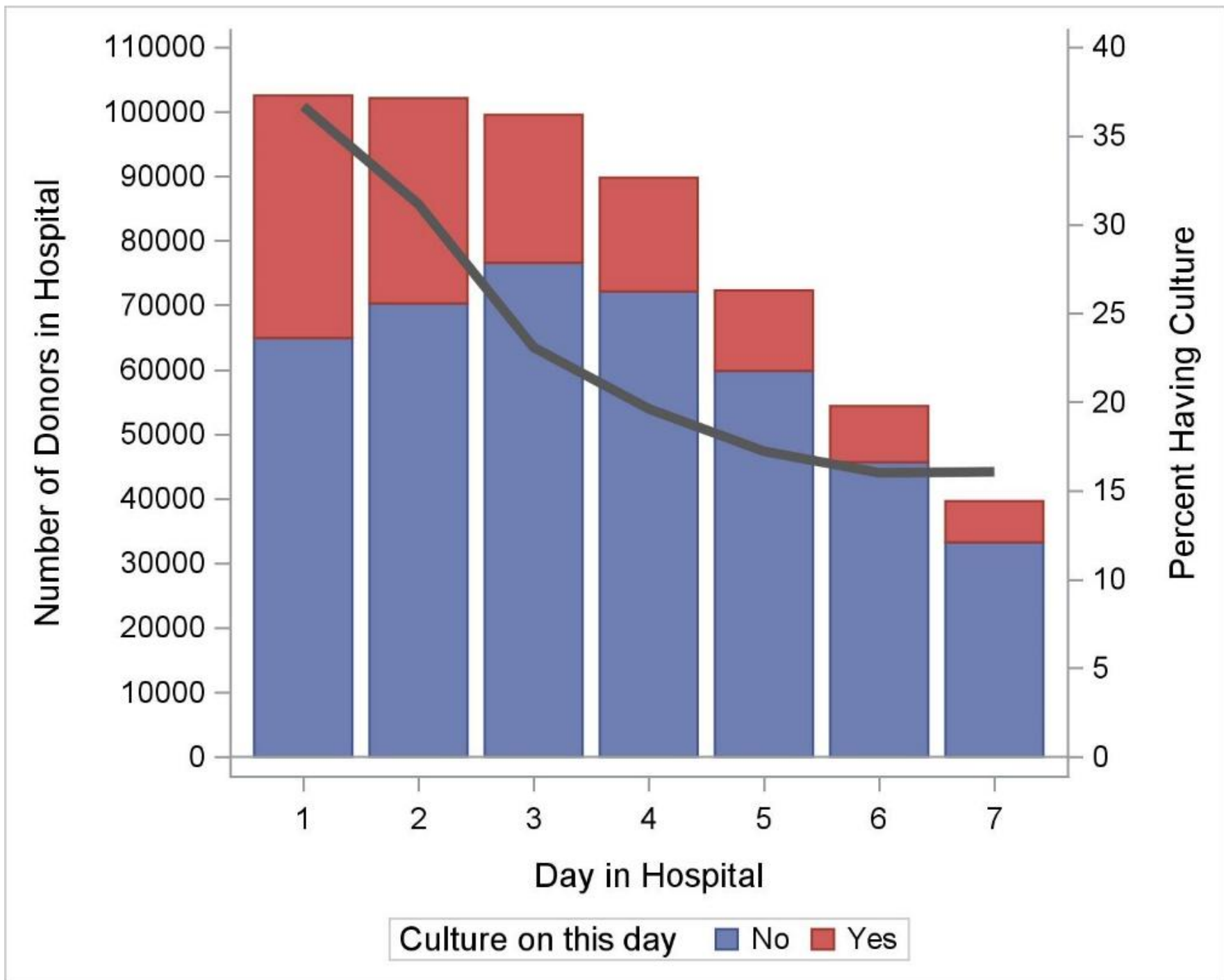

The gray line represents the observed percent having culture by day with corresponding y axis on the right side of the graph.

Figure S3. Total Number of blood culture and percentage of positive blood culture results from admission day 1 to day 7.

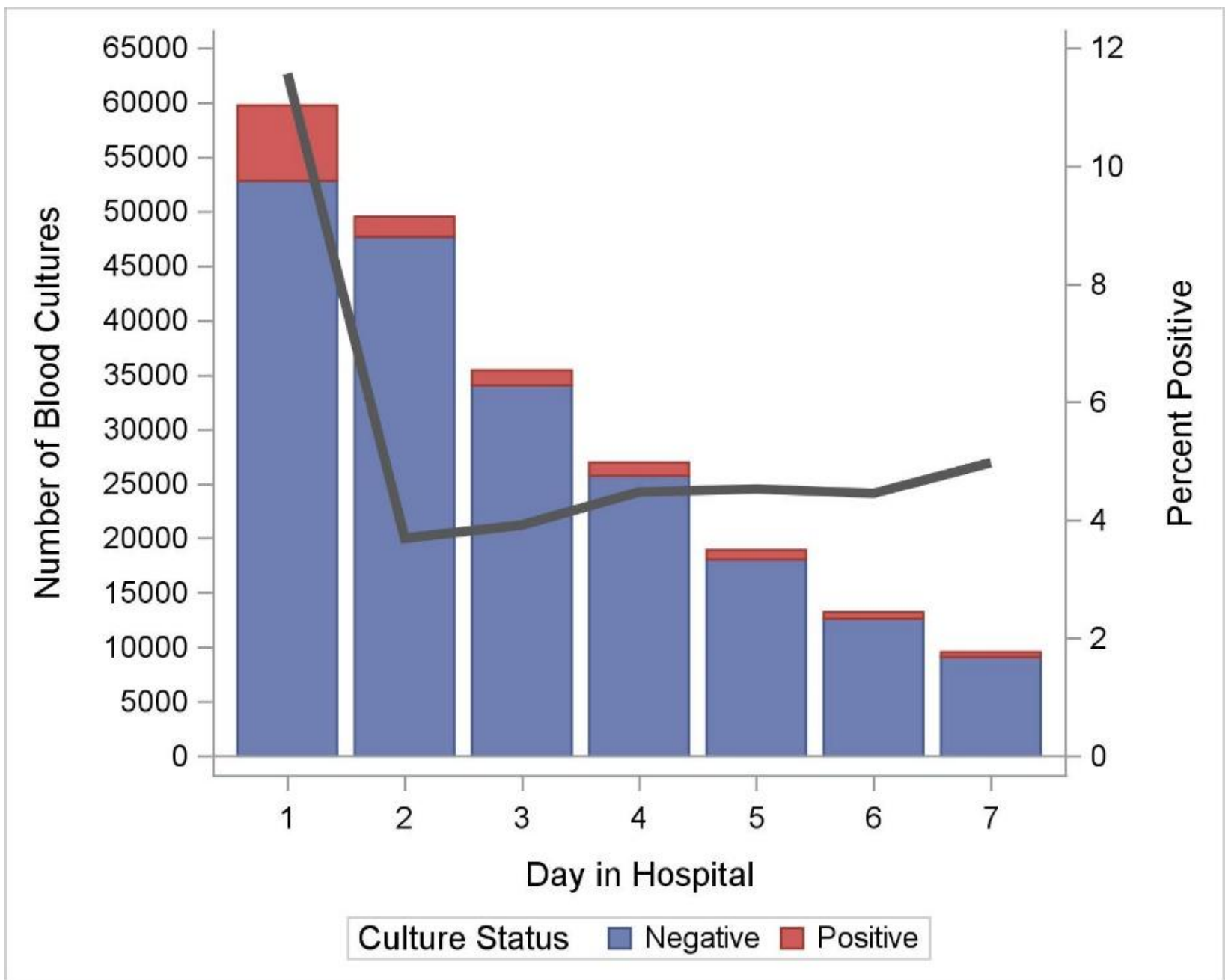

The gray line represents the observed percent positive by day with corresponding y axis on the right side of the graph.

Supplementary references

1. Wholley CL. Guidance on Explaining Risk Related to Use of U.S. PHS Increased Risk Donor Organs When Considering Organ Offers. OPTN/UNOS Public Comment Proposal. Accessed March 1, 2026. https://www.hrsa.gov/optn/policies-bylaws/public-comment/guidance-phs-increased-risk-donor-organs.

2. Social Deprivation Index (SDI). Accessed November 1, 2025. https://www.graham-center.org/maps-data-tools/social-deprivation-index.html.
